# Explainable artificial intelligence models for predicting risk of suicide using health administrative data in Quebec

**DOI:** 10.1371/journal.pone.0301117

**Published:** 2024-04-03

**Authors:** Fatemeh Gholi Zadeh Kharrat, Christian Gagne, Alain Lesage, Geneviève Gariépy, Jean-François Pelletier, Camille Brousseau-Paradis, Louis Rochette, Eric Pelletier, Pascale Lévesque, Mada Mohammed, JianLi Wang

**Affiliations:** 1 Institut Intelligence et Données (IID), Université Laval, Québec, Québec, Canada; 2 Institut National de Santé Publique du Québec (INSPQ), Québec, Québec, Canada; 3 Department of Psychiatry and Addiction, Université de Montréal, Montreal, QC, Canada; 4 Centre de Recherche de l’Institut Universitaire en Santé Mentale de Montréal, Québec, Canada; 5 Centre for Surveillance and Applied Research, Health Promotion and Chronic Disease Prevention Branch, Public Health Agency of Canada, Ottawa, Canada; 6 Department of Social and Preventive Medicine, School of Public Health, University of Montreal, Montreal, Canada; 7 Montreal Mental Health University Institute Research Center, Montreal, Canada; 8 Department of Community Health and Epidemiology, Faculty of Medicine, Dalhousie University, Halifax, Canada; University of Connecticut Health Center: UConn Health, UNITED STATES

## Abstract

Suicide is a complex, multidimensional event, and a significant challenge for prevention globally. Artificial intelligence (AI) and machine learning (ML) have emerged to harness large-scale datasets to enhance risk detection. In order to trust and act upon the predictions made with ML, more intuitive user interfaces must be validated. Thus, Interpretable AI is one of the crucial directions which could allow policy and decision makers to make reasonable and data-driven decisions that can ultimately lead to better mental health services planning and suicide prevention. This research aimed to develop sex-specific ML models for predicting the population risk of suicide and to interpret the models. Data were from the Quebec Integrated Chronic Disease Surveillance System (QICDSS), covering up to 98% of the population in the province of Quebec and containing data for over 20,000 suicides between 2002 and 2019. We employed a case-control study design. Individuals were considered cases if they were aged 15+ and had died from suicide between January 1st, 2002, and December 31st, 2019 (n = 18339). Controls were a random sample of 1% of the Quebec population aged 15+ of each year, who were alive on December 31st of each year, from 2002 to 2019 (n = 1,307,370). We included 103 features, including individual, programmatic, systemic, and community factors, measured up to five years prior to the suicide events. We trained and then validated the sex-specific predictive risk model using supervised ML algorithms, including Logistic Regression (LR), Random Forest (RF), Extreme Gradient Boosting (XGBoost) and Multilayer perceptron (MLP). We computed operating characteristics, including sensitivity, specificity, and Positive Predictive Value (PPV). We then generated receiver operating characteristic (ROC) curves to predict suicides and calibration measures. For interpretability, Shapley Additive Explanations (SHAP) was used with the global explanation to determine how much the input features contribute to the models’ output and the largest absolute coefficients. The best sensitivity was 0.38 with logistic regression for males and 0.47 with MLP for females; the XGBoost Classifier with 0.25 for males and 0.19 for females had the best precision (PPV). This study demonstrated the useful potential of explainable AI models as tools for decision-making and population-level suicide prevention actions. The ML models included individual, programmatic, systemic, and community levels variables available routinely to decision makers and planners in a public managed care system. Caution shall be exercised in the interpretation of variables associated in a predictive model since they are not causal, and other designs are required to establish the value of individual treatments. The next steps are to produce an intuitive user interface for decision makers, planners and other stakeholders like clinicians or representatives of families and people with live experience of suicidal behaviors or death by suicide. For example, how variations in the quality of local area primary care programs for depression or substance use disorders or increased in regional mental health and addiction budgets would lower suicide rates.

## Introduction

Suicide is a complex public health issue and one of the leading causes of death worldwide. It remains difficult to predict suicidal behaviors accurately and consistently despite increased awareness of suicide as a significant cause of avoidable death [1]. Every suicide is a disaster that affects families, neighborhoods, and entire countries and that has long-lasting effects on the people left behind [2]. In Quebec, suicide takes the lives of more than a thousand individuals per year [3] for a population of 8.7 million inhabitants Every day, approximately 3 Quebecers tragically take their lives. However, suicide remains a rare event with 2017 age-standardized mortality rate by suicide of 11.4 per 100,000 overall and 17.2 and 5.8 per 100,000 in males and females, respectively [4]. In Canada, it is approximated that for each death by suicide, there are 25–30 additional suicide attempts, many of which result in emergency department visits, immediate hospitalization, and/or mental health facility admissions in the past year [5–7].

Mental health conditions, such as depression, bipolar disorder, schizophrenia, and substance abuse disorders, have consistently been shown to be prevalent among those who die by suicide [8]. Mental and substance use disorders are found in 95% of suicide cases, and the population attributable fraction for mental disorder were ranged from 47–74% [9]. Systematic audits which consist of bottom-up evidence, indicated that deficits in primary care mental health or addiction services can be identified in over 80% of all suicides [10, 11]. From a populational perspective, suicide may be related to factors at the individual (e.g., mental disorders), programmatic (e.g., visits to Emergency Departments; adequacy of follow up for depression in the local area primary care), system (e.g., per capita regional budget for substance use disorders), and community level (e.g., neighborhood social deprivation) [12].

The growth of data and machine learning (ML) has the potential of impacting deeply all aspects of healthcare, among other domains. ML is a branch of artificial intelligence (AI) that relies on statistical and probabilistic techniques to automatically learn patterns and enhance performance on specific tasks through exposure to data [13]. Nevertheless, caution must be taken in medical and public health applications as "black-box" models that automatically improve via experience can pose potential risks and, as such, hinder its adoption. Close supervision is required to ensure safety and accuracy. However, such techniques are relevant as they offer powerful models that can capture interactions between potentially correlated risk factors and suicide, which can be difficult to achieve at adequate levels with conventional statistical methods [14]. Recent research has revealed a range of advantages of ML that can assist in detecting, diagnosing, and treating mental health problems, and predicting the suicidal behaviours level risk in populations [14–18]. For example, Gradus et al. [17] created sex-specific ML models utilizing data from eight Danish national health and social registries, encompassing over 90% of the Danish populace. They found that sex-specific suicide risk in Denmark was significantly associated with factors linked to substance use disorders (SUDs), such as alcohol-related disorders and prior poisoning. About 20% of men and women who died by suicide had SUDs. However, whether risk factors for suicide differ between the high-risk SUD population and the general population is an area that remains mostly unexplored. Kessler et al. [18]. developed ML models to predict suicide risk among USA army soldiers after hospital stays and in the Veterans Health Administration system. Even though this study has been helpful, the results from US army members and veterans may not be generalizable to the larger community of patients admitted to psychiatric hospitals. Furthermore, it is unclear whether men and women have different suicide risk profiles after being admitted to mental hospitals. Walsh et al. [19] constructed a predictive model for adolescents using electronic health record data and the model performed well in a limited Southern US population. Nevertheless, a more comprehensive risk predictive models that considers a broader range of clinical factors beyond mental health comorbidities and medications and generates quantifiable risk scores to determine patients’ suicide risk levels is necessary.

Despite the advancements in AI, a significant impediment to adopting AI systems in healthcare is that many are seen as "black-boxes” by stakeholders and decision- makers referring to their lack of interpretability [20]. When we refer to an algorithm as a “black box,” we mean that the estimated function that relates inputs to outputs is not understandable at an ordinary human level; for instance, the function that relies on many parameters, complicated parameter combinations, or nonlinear parameter transformations. Explainable Artificial Intelligence (XAI) proposes shifting toward more transparent AI to address this issue. XAI typically refers to post hoc analyses and techniques used to understand previously trained "black-box models" or their predictions [21–23]. It is significant because it is necessary to understand the causality of learned representations for decision support and helps assess whether the model is considering the right features while making a specific prediction [24–26]. XAI methods can provide various explanations, such as global, local, contrastive, what-if, counterfactual, and example-based [26, 27]. Each approach to interpretability can be used for real-world problems based on the characteristics of the environment where we would like to use a specific approach [28]. As part of XAI, global explanations techniques provide top-down mental representation of the AI model’s behavior, typically in the form of visual charts, mathematical formulae, or model graphs [27]. Among XAI methods, SHapley Additive explanations (SHAP) has emerged as a prominent choice for several reasons. First, it provides a unified interpretability framework that can be applied to diverse ML models without requiring model-specific modifications. This versatility makes the SHAP applicable across various domains. Second, SHAP offers local and global interpretability, allowing insights into individual feature contributions and model behavior. Its solid theoretical foundation in game theory ensures fairness and consistency in attributing the feature importance. Lastly, SHAP facilitates clear and intuitive visualizations, making explanations easily understandable and facilitating stakeholder collaboration [29]. Considering the constraints imposed by the confidentiality of our dataset, we chose to employ SHAP as a global explanation technique for our project because it has proven to be effective in revealing the behavior of ML models.

Studies have consistently shown that suicide affects more men than women [30–32]. Research has found a correlation between traditional masculine traits, such as independence, assertiveness, leadership, and dominance, and an increased risk of suicidal thoughts in middle-aged men [33–39] However, while men are more likely to die by suicide, women are more likely to engage in suicidal behaviors and deliberate self-harm. This is known as the "gender paradox" of suicide, which has been consistently shown across various studies [40–42]. Moreover, retrospective cohorts of the Australian and white American populations have revealed that suicide rates progressively increase throughout life for males, whereas females have their most elevated rates between 35–54 and 45–54 years, respectively [43, 44]. Didier et al. [45] have shown that men face a higher risk of suicide than women in most nations, as evidenced by their study on the gender paradox in suicidal behavior and its impact on the suicidal process. Considering the differences in the prevalence, risk factors, and protective factors for suicide between males and females, this study aims to develop sex-specific suicide risk prediction supervised ML models and apply a post-hoc global explanation approach to interpret the findings.

## Materials and methods

### Ethical approval

This project was approved by the Ethics committee of both the Dalhousie University and of Université Laval. Access to the QICDSS was approved by government bodies, the Public Health Ethics Committee, and the Commission d’accès à l’information du Québec for chronic disease surveillance purposes (Blais C, Jean S, Sirois C, Rochette L, Plante C, Larocque I, Doucet M, Ruel G, Simard M, Gamache P (2014) Quebec integrated chronic disease surveillance system (QICDSS), an innovative approach. Chronic Dis Inj Can 34(4):226–235) Informed consent was not required in the context of register-based studies that use anonymized data. This study was performed in line with the principles of the 1964 Helsinki Declaration.

### Data sources

The data source for this study, the Quebec Integrated Chronic Disease Surveillance System (QICDSS), was accessed in 1-11-2020, covering up to 98% of the province of Quebec’s population and containing data for over 20,000 suicides between 2002 and 2019. The QICDSS consists of five linked databases, namely the health insurance registry (demographic information), the physician billing database (all medical fee-for-service acts billed to the Régie de l’assurance maladie du Québec, including diagnoses), the hospitalization database (including primary and secondary diagnoses based on the International Classification of Diseases, 9th revision (ICD-9) until April 2006 and ICD-10 after that), the prescription claim database for those covered by the public drug plan and the vital statistics death database. A detailed description of the QICDSS is available elsewhere [46]. Individual occurrences of suicide within the general population are comparatively infrequent when contrasted with other causes of death [47]. we used a case-control study design to unravel the nuanced factors associated with suicide.

The training dataset included individuals aged 15 or older who died from suicide between January 1st, 2002, and December 31st, 2010 (n = 9,440) as cases, and a random sample of 1% of the Quebec population aged 15 or older who were alive on December 31st of each year, from 2002 to 2010, to track account for potential population changes over time (n = 661,780) as controls. Similarly, for the test dataset, individuals aged 15 or older who died from suicide between January 1st, 2011, and December 31st, 2019 (n = 8,899) were considered cases. Controls were selected as a random sample of 1% of the Quebec population aged 15 or older who were alive on December 31st of each year from 2011 to 2019 (n = 645,590). In order to maximize the variability of predictors, cases and controls were not matched.

### Features

We included 103 individual, programmatic, systemic, and community features measured starting five years (60 months) before the suicide events—see “S1 Table: List of variables”. Some features (e.g., diagnoses, utilization service) were dummy coded to create time intervals ranging from 0–6 to 0–60 months before the first day of the suicide month. Missing data is common in real-world big data sources. To handle missing features in our dataset, we employed several imputation techniques based on the type of variable. For continuous (numeric) variables, mean and median imputation methods were utilized, while mode imputation was used for categorical (nominal) variables. Additionally, for categorical variables with multiple categories, we used one-hot encoding [48] to convert them into binary form.

### ML models

We used Python for the model development and analysis. Model development and evaluation were accomplished in three phases.

#### Phase 1: Model building

We developed sex-specific supervised ML models. The term "sex-specific" refers to our approach of training the model on the complete dataset but conducting separate evaluations based on sex (male and female) during the testing phase. It is important to note that we did not create separate models for each sex but rather utilized the same model to assess performance within each group. The values that were produced by the models during training were compared to target values, providing feedback to the learner. The train-test split method was used [49] to estimate the performance of our ML models. For the final model, we used data from 2002 to 2010 with 9440 suicide cases (7234 males and 2206 females) and 661,780 controls for a train set, and from 2011 to 2019 with 8899 cases (6713 males and 2186 females) and 645,590 controls for a test set. The test set was kept aside for the model’s final evaluation, while the train set was used to train the models. We can quickly and effectively test our trained models by using data they have never seen before.

**Fig 1:**
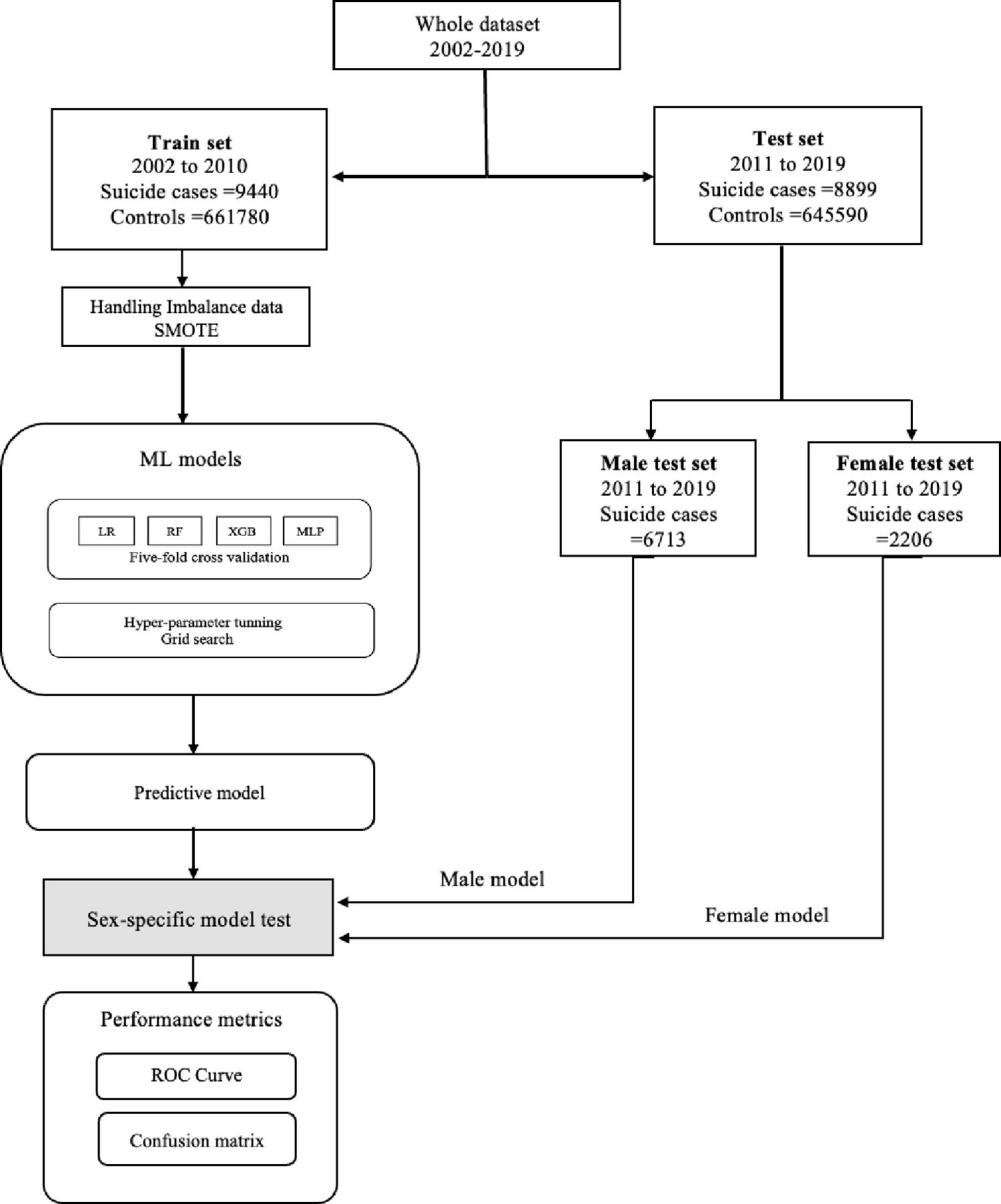
Schematic representation of the development of the prediction model. SMOTE: Synthetic Minority Over-Sampling Technique.

We trained supervised ML classifications with Logistic Regression (LR), Random Forest (RF), Extreme Gradient Boosting (XGBoost), and Multilayer Perceptron (MLP) with an optimized model architecture. LR assumes a linear relationship between the logit of the outcome and predictor variables and has been extensively utilized in ML research on suicide predictions [18, 19]. RF is a tree-based algorithm that ensembles of many decision trees based on bootstrapped samples and aggregates votes (predicted class) from each tree. It enhances the classification tree by considering a random subspace of predictors when building a tree and by creating a diverse set of trees that contribute to classification performance [50]. The XGBoost method is a variant of the gradient boosting algorithm, which minimizes errors by applying the gradient descent method in a Boosting algorithm combining several weak learners [51]. MLP is a neural network classifier consisting of feedforward networks with dense, all-to-all connections between layers. The network uses nonlinear transformations to learn high-level abstractions in the data to build the predictive model [52, 53]. The optimal hyperparameter for models was found through a five-fold cross-validated grid—see “S2 Table: Hyperparameters”. Then, a model was built using the learned parameters for the whole training set, and predictions were made on the testing set, which was never utilized for model selection or parameter tuning. Schematic representation of the development of the prediction model has shown in (Fig 1).

*Handling data imbalance*. The dataset was imbalanced as cases of suicide were rare relative to controls. Classification would be biased towards the positive class if the imbalance is not handled. This can result in a falsely perceived positive effect on the model’s accuracy. To address this problem, we used the Synthetic Minority Over-sampling Technique (SMOTE) [54].

SMOTE not only rectifies the class distribution imbalance but also introduces diversity and variability into the minority class, which can be advantageous for model training. By oversampling the minority class, SMOTE avoids discarding potentially valuable information. The application of SMOTE was performed solely on the training set to enhance class balance, while keeping the test set intact to ensure a realistic test distribution is preserved.

#### Phase 2: Model evaluation

The discriminatory performances were assessed using the Receiver Operating Characteristics (ROC), the Area Under Curve (AUC), and various operating characteristics, including sensitivity, specificity, and positive predictive value (PPV) with an adjusted threshold. Threshold adjustment was a meticulous process in which we extensively evaluated the trade-offs between sensitivity, specificity, and PPV. We conducted a comprehensive evaluation process to determine the optimal adjusted threshold, including techniques such as ROC analysis and precision-recall curves. We aimed to find a perfect equilibrium that maximized the identification of near-suicide cases while minimizing false positives. The model calibration was assessed by using Temperature Scaling (TS). TS is a post-processing technique that uses a single scalar to smooth the softmax output and regularize the entropy [55, 56] with the following equation:

TS(y^)=ezT∑jezjT (1)

where $\hat{y}$ is the prediction and *z* is the logit vector by a learned scaler parameter T.

The calibrated probabilities by TS can approximately represent the confidence score of model predictions, which means the alignment between the observed accuracy distribution and the predicted probabilities. These confidence scores should match the true correctness likelihood [56]. The reliability diagram is a simple way to visualize calibration accuracy as a function of confidence [57]. The confidence score and accuracy should match when a model is calibrated. For instance, if the model predicts 0.8 for 100 examples in the test set, the accuracy over those 100 examples should be very close to 80%. If this is the case, we say that the model is well calibrated.

#### Phase 3: Explainability

To examine the contribution of each feature to the predictive model output, we used the SHapley Additive explanations (SHAP) [58]. SHAP is based on coalitional game theory and can be used to explain the output of any ML models. SHAP values are calculated as a measure of the impact of each feature by comparing the prediction results with and without that feature [58, 59]. The Shapley values are computed by taking the average of the marginal contribution of each feature value across all possible values in the feature space [58]. The terms Shapley values and SHAP values are frequently used interchangeably. Technically speaking, this is incorrect as Shapley values represent the theory, and SHAP values are a specific implementation for calculating Shapley values.

*SHAP summary plot*. The summary plot is demonstrated to combine feature importance with feature effects. The position on the x-axis is determined by Shapley value, and the y-axis by the feature. The colors of the dots indicate if the value of the corresponding feature is high (usually in red) or low (usually in blue) [58]. Given a coalitional game (*N*, *v*), the Shapley value of player (feature) *i* is expressed as:

$$\emptyset_{i}(N,v)=\frac{1}{N!}\sum_{S\subseteq N\backslash \{i\}}|S|!(|N|-|S|-1)![(v(S\cup \{i\})-v(S))]$$ (2)

Where ∅_*i*_(*N*, *v*) is denoted the Shapley value ∅ of *i* in a subset (or coalition) *N* and a value *v*. For example, how much feature (i.e., age) contributed to the prediction risk of suicide. *S*⊆*N*\{*i*} means that the elements of *S* will be included in *N* but not *i*. If *N = {age*, *sex*, *location}*, *S* could be *{sex*, *location}* with *i = age*. If we compare *S* with and without “age”, we will find a different marginal contribution value *v*.

The weighing factor |*S*|!(|*N*|−|*S*|−1)! counts the number of ways the subset S can be permuted, [*v*(*S*∪{*i*})−*v*(*S*)] calculates the difference of the value *v* of a subset *S* of *N* not containing *i* with the same subset *i*. Σ_*s*⊆*N*{*i*}_ sum over all possible sets $S,\;\frac{1}{N!}$ finally, sum over all possible sets *S* and obtain an average by dividing by *N*!, the number of players participating in the game, i.e., the number of features that we have in total.

XAI and feature contributions were presented using the coefficients and Shapley values, this paper computes Shapley value using the SHAP package in Python.

## Results

### Description of the sample

The suicide cases included 7,234 (76.63%) men and 2,206 (23.36%) women. People who died by suicide were slightly older than the non-suicide group for (mean [SD] of 45.3 [16.0] vs 44.3 [17.6] years) and similar for women (46.2 [15.3] vs 46.4 [18.9] years). Regarding material and social deprivation, the suicide cases lived in more deprived areas. Tables 1 and 2 provide an overview of the demographic characteristics in the study population for the training and test sets, respectively.

**Table 1:** Socio-demographic characteristic train set (2002 to 2010).

Variables	Control				Suicide
	Total n = 661780				Total n = 9440
	N (%)				N (%)
Age *					
15–39		263362(39.79)	3289(34.84)		
40–59		244541(36.95)	4504(47.71)		
≥ 60		153877(23.25)	1647(17.44)		
**Sex**					
Male		322361(48.71)	7234(76.63)		
Female		339419(51.28)	2206(23.36)		
Urbanicity **					
Rural Town		134218(20.28)	2659(28.16)		
Social Deprivation score***					
1 (most privileged)		124334(18.78)	1507(15.96)		
2		125632(18.98)	1771(18.76)		
3		127401(19.25)	1674(17.73)		
4		128279 (19.38)	1896(20.08)		
5 (most deprived)		128857(19.47)	2207(23.37)		
Material Deprivation score***					
1 (most privileged)		126110 (19.05)	1275(13.50)		
2		126600 (19.13)	1538(16.29)		
3		127448(19.25)	1853(19.62)		
4		128052(19.34)	2037(21.57)		
5 (most deprived)		126293(19.08)	2352(24.91)		

* In the dataset, Age (without missing) is a continuous variable used in the ML model; however, it is shown in this table as a categorical variable for better representation.

**Missing: Control group: n = 5823 (0.87%); Suicide group: n = 40 (0.42%)

***Missing: Control group: n = 27277 (4.12%); Suicide group: n = 385 (4.07%)

**Table 2:** Socio-demographic Characteristic Sex-specific Test Set (2011 to 2019).

	Men		Women	
Variables	Control	Suicide	Control	Suicide
	Total n = 316574	Total n = 6713	Total n = 329016	Total n = 2186
	N (%)	N (%)	N (%)	N (%)
Age*				
15–39	122712 (38.76)	1948 (29.01)	120116 (36.50)	612 (27.99)
40–59	108687 (34.33)	2998 (44.65)	108740 (33.05)	1043 (47.71)
≥ 60	85175 (26.90)	1767 (26.32)	100160 (30.44)	531 (24.29)
Urbanicity **				
Rural Town	62682 (19.80)	1962 (29.22)	60449 (18.37)	478 (21.86)
Social Deprivation score***				
1 (most privileged)	60096 (18.98)	1167 (17.38)	59127 (17.97)	310 (14.18)
2	60283 (19.04)	1323 (19.70)	60002 (18.23)	339 (15.50)
3	59937 (18.93)	1223 (18.21)	61491 (18.68)	358 (16.37)
4	58681 (18.53)	1162 (17.30)	62807 (19.08)	427 (19.53)
5 (most deprived)	58620 (18.51)	1427 (21.25)	63683 (19.35)	592 (27.08)
Material Deprivation score ***				
1 (most privileged)	58609 (18.51)	875 (13.03)	62815 (19.09)	323(14.77)
2	58650 (18.52)	1034 (15.40)	61194 (18.59)	365 (16.69)
3	60167 (19.00)	1274 (18.97)	61428 (18.67)	378 (17.29)
4	60102 (18.98)	1450 (21.59)	61349 (18.64)	449 (20.53)
5 (most deprived)	60089 (18.98)	1669 (24.86)	60324 (18.33)	511 (23.37)

* In the dataset, Age (without missing) is a continuous variable used in the ML model; however, it is shown in this table as a categorical variable for better representation.

**Missing: Men: Control group: n = 4611 (1.45%); Suicide group: n = 30 (0.44%); Women: Control group: n = 3196 (0.97%); Suicide group: n = 6 (0.22%).

***Missing: Men: Control group: n = 18957 (5.98%); Suicide group: n = 411 (6.12%); Women: Control group: n = 21906 (6.65%); Suicide group: n = 160 (7.31%).

### Model performance

**Fig 2:**
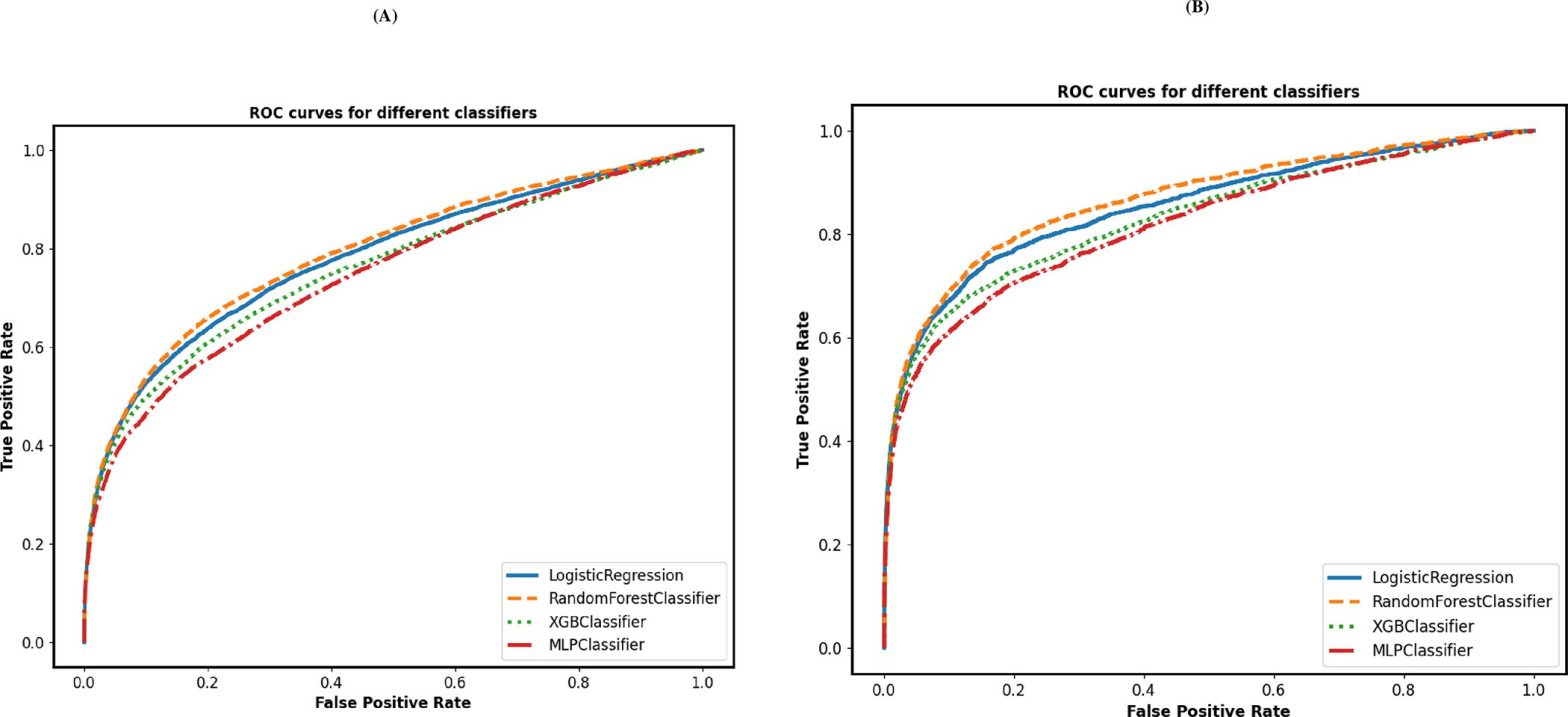
Area Under the Receiver Characteristics Curve (AUROC). Discriminatory performance of models A: the area under the receiver characteristics curve (AUROC) of men; B: the area under the receiver characteristics curve (AUROC) of women.

Table 3 and Fig 2 present the classification performance of the four models for predicting the risk of suicide. Assessed graphically with a reliability diagram before and after calibration is shown in “S1 Fig: Calibration plots “.

**Table 3:** Models performance.

Without SMOT (Imbalanced)				
Men model	Precision	Sensitivity	Specificity	AUC
LR	0.60	0.08	1.00	0.79
RF	0.65	0.07	1.00	0.79
XGBoost	0.63	0.09	1.00	0.80
MLP	0.60	0.08	1.00	0.75
**Women model**				
LR	0.41	0.05	1.00	0.87
RF	0.67	0.07	1.00	0.85
XGBoost	0.52	0.09	1.00	0.83
MLP	0.42	0.06	1.00	0.87
**With SMOT (balanced)**				
**Men model**	**Precision**	**Sensitivity**	**Specificity**	**AUC**
LR	0.19	0.38	0.97	0.79
RF	0.21	0.36	0.97	0.79
XGBoost	0.25	0.31	0.98	0.76
MLP	0.20	0.32	0.97	0.76
**Women model**				
LR	0.16	0.43	0.99	0.86
RF	0.15	0.45	0.98	0.87
XGBoost	0.19	0.40	0.99	0.84
MLP	0.11	0.47	0.97	0.83

LR: Logistic Regression; RF: Random Forest; XGBoost: Extreme Gradient Boosting; MLP Multilayer perceptron

The men’s model showed that the accuracy rate was the highest for XGBoost (0.97), followed by RF (0.96), MLP (0.96) and LR (0.95). The four models showed a sensitivity of 0.31–0.38, meaning that the models correctly identified approximately 31–38% of men who died by suicide. Other metrics showed a specificity of 0.97–0.98 and a PPV of 0.20–0.25. The cross-validated AUC was the highest with RF, and LR (0.79), and the lowest with XGBoost and MLP (0.76). Overall, LR appeared to provide more accurate classification results than the other algorithms when predicting suicide in men.

The women’s model showed an accuracy for XGBoost (0.98), RF (0.98), MLP (0.97) and LR (0.98), and a sensitivity of 0.40–0.47 across the four classification models, meaning that the models correctly identified approximately 40–47% of women who died by suicide. Other metrics showed a specificity of 0.97–0.99 and PPV of 0.11–0.19. The cross-validated AUCs was the highest with RF (0.87) and lowest with MLP (0.83). Overall, the women’s model showed better performance compared to the men’s model.

### Feature importance

#### Absolute coefficients

**Fig 3:**
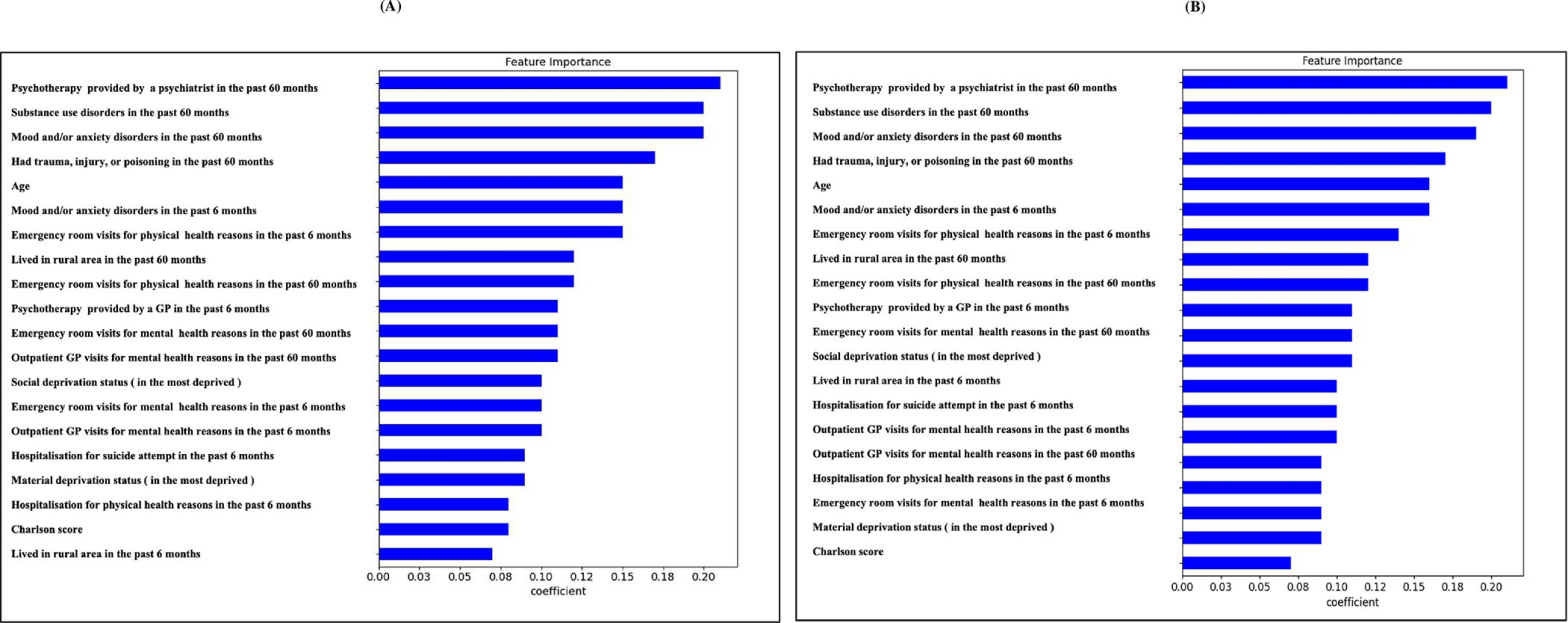
Feature importance of the logistic regression model. The 20 most important features in the LR model with sex-specific A: men model and B: women model.

Fig 3A and 3B illustrate the 20 features with the biggest absolute coefficients using LR prioritized differently based on the sex-specific models. Psychotherapy visits with a psychiatrist in the past 60 months were highly associated with the risk of suicide, followed by mood and anxiety disorders in the past sixty months, substance use disorders in the past 60 months, age, and non-intentional trauma in the past 60 months.

#### XAI with SHAP

**Fig 4:**
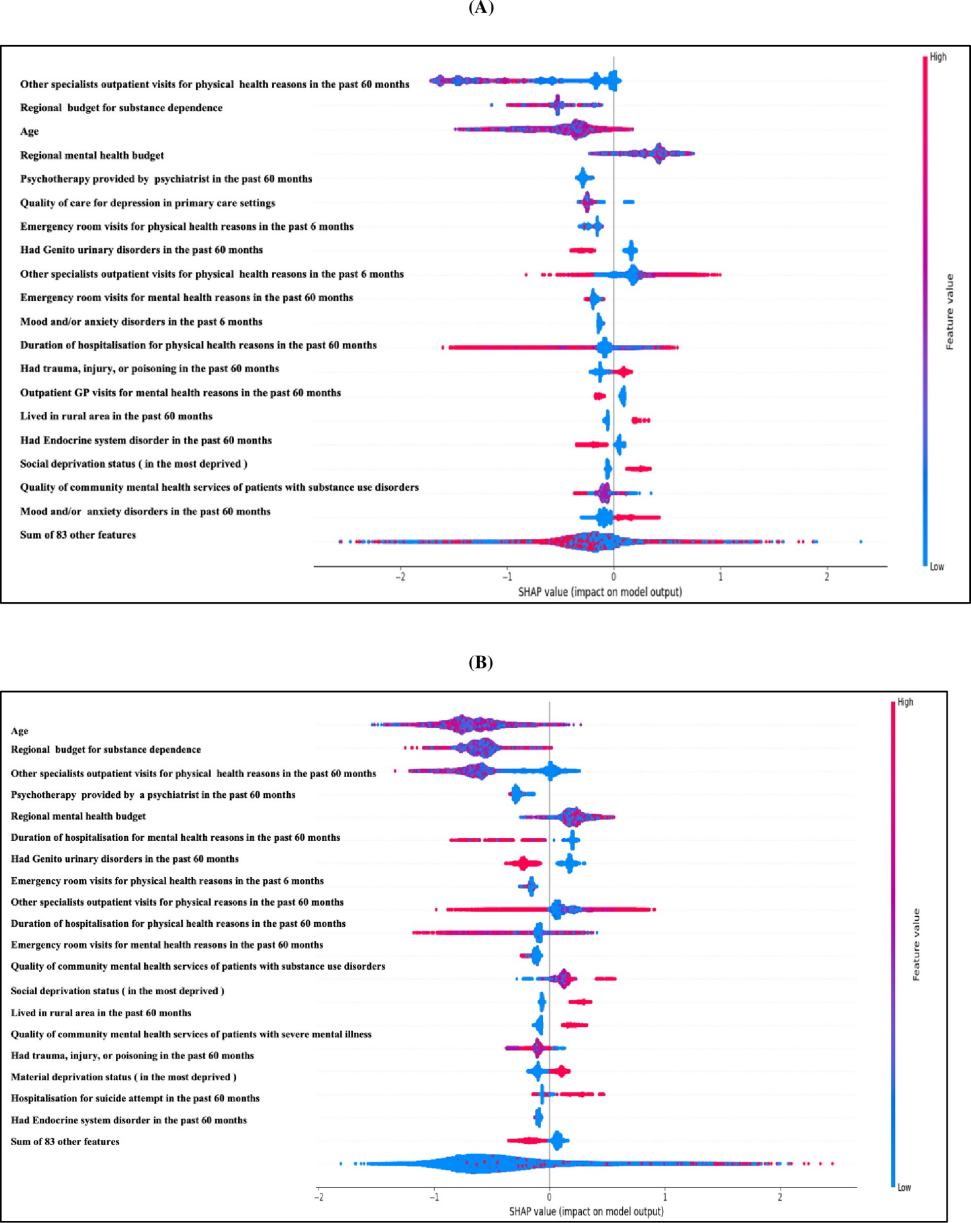
SHAP summary plot for the XGBoost-based suicide prediction model. The 20 most important SHAP-value with sex-specific A: men model and B: women model.

The SHAP feature importance values were used to identify the impact of each feature on the prediction of suicide risk. The top 20 features of the model are listed on the y-axis in Fig 4A and 4B and are ranked from most important to least important for men and women, respectively. The variables at the top considerably influenced the prediction outcomes, whereas the variables at the bottom had less of an effect on the result. From Fig 4A and 4B, it could be inferred that the top 5 features for predicting the risk of suicide for both (men and women) were age, specialist outpatient visits for physical disorders in the past sixty months, regional mental health budget, regional dependence budget, and psychotherapy visits with a psychiatrist in the past 60 months. Examples of Fig 4A are discussed below to give the readers a glimpse of the interpretation. One example we can pick from Fig 4A is that male patients with non-intentional trauma would have an increased risk of suicide as denoted with red color, which corresponds to the positive values in the x-axis. Another case from Fig 4B is for woman with a high positive value (red color) for social and material deprivation, which denoted an increased suicide risk (value corresponding to the x-axis). In other words, social and material deprivation was present in many cases of woman suicide.

## Discussion

In this study, we demonstrated the feasibility of predicting the risk of suicide with health administrative data using ML techniques. The main objective of the present study was to examine the supervised ML approach for the sex-specific suicide risk prediction model. Our ML classification achieved 0.76–0.79 AUC in men models, and accuracy and 0.83–0.87 AUC in women models. The weights in the LR model were used to identify the parameters and attributes that are globally or locally significant for accurate prediction. The important features found with absolute coefficients were consistent with previous research on their associations with suicide risk at the individual, programmatic and community level [60–64]. Considering the 10 most significant associations of in Fig 3, a first set of individual variables pertain to mental disorders and substance use disorders are well established risk factor for suicide [63–67]. A second set of variables are contact with primary care physicians and specialist mental health care, and Emergency Departments (ED) visits, which are indicative of greater need for care, but not always receiving adequate care. Systematic audits have shown that half of suicide cases were in contact in the last year with the ED, 50% in contact with a general practitioner and 25% with a psychiatrist, and deficits have consistently been found in the 3 areas, i.e., coordination of specialist mental health and specialist addiction services at the ED; poor access to referral by general practitioners to specialist mental health or addiction consultation; poor access to mobile crisis resolution teams in support of outpatient services [10]. The increased risk of suicide among patients presenting for non-intentional trauma at the ED may be due to alcohol abuse. This indicates that more systematic suicide risk assessment would be warranted for this group. An increased suicide risk was also found among outpatients receiving more psychotherapy, likely reflecting the severity of their mental health condition. A third set of variables (age; rural areas) can be described as individual and community levels variables which are significantly associated with suicide [60–62]. Finally, the strong link with regional addiction or mental health budget echoed in the very recent Chief Coroner public enquiry on suicide based on a series of individual cases audit and their aggregation, suggested an increase in the public managed care health and social services ministry budget for mental health and addiction services [68].

Another objective of this study was to provide better insights into ML models, given that most ML models are usually sophisticated and black-boxes are difficult to comprehend. For instance, the non-linear model, such as RF, XGBoost, and neural networks, perform well in accurately classifying the data and making predictions but they are difficult to understand regarding how they make their decision from the raw models and as such to gain trust on their predictions. XAI aims at providing some explanations on the elements leading to decision over such black-box models [69]. In the current study, SHAP was used for achieving explainability by determining the contribution of each variable in the model. SHAP values offer different interpretations, such as global interpretability, which highlighted the significance of every indicator and local interpretability, which determines SHAP values specifically for each instance. This significantly increases the transparency and helps explain case prediction and major decision contributors [58, 70]. For SHAP values, various visualizations can be employed to help model interpretation and provide explanations for the results, such as Force plots for single predictions, the summary plot of all SHAP values, and dependence plots [71]. In this study, we used the summary plot to offer a broader view of the importance of each feature that had a positive or negative impact on the suicide.

In the SHAP summary plot, the top variables, including age, specialist outpatient visits for physical disorders in the past 60 months, regional mental budget, regional dependence budget, psychiatric outpatient psychotherapy in the past 60 months, and quality of local area primary care for depression, provided the insight into the local area programs where the individual lives [12, 72]. The association with other specialist physical health visits may seem to be counterintuitive, but it is related to the deficits in identifying suicide risk and consultation with physical mental health or addiction services, as evidenced by at least one case [10]. Similarly, while capturing the quality of psychiatric outpatient visits with potentially effective and relevant treatments like psychotherapy, the modeling indicated that the low number of psychotherapy visits with a psychiatrist in the past 60 months was associated with lower risk, but not the local area quality of primary care physician practices for depression where the individual was living associated with lower risk.

The former variable does not allow disentanglement of severity with the quality of the intervention in the association, whereas the latter is more indicative of the local care environment where the individual may have accessed timely care. The adequate balance of local primary care and specialist mental health care has been evidenced in Finland by Pirkola et al., 2009 [73]. In addition to continuous suicide risk evaluation of outpatient psychotherapy patients, the identified variable may indicate a need for more publicly funded private practice psychiatrists in the areas where there is a relative shortage of mental health professionals in emergency departments, hospitals, and community care [74]. Another important system-level variable is the regional dependence budget [75], which funds addiction programs in Quebec at approximately $150 million annually, with $1.5 billion allocated for specialist mental health care [76].

Substance use disorders explain over 25% of the populational attributable risk of suicide [77]. Registry-based studies indicated a 3 to 4-fold increase in mortality of people diagnosed with substance use disorders, including cancer, cardiovascular diseases, suicide and accidental deaths [78]. Tondo et al.2006 [79] demonstrated that association between mental health expenditures and suicide rates in the United States. However, there is a larger health economics literature linking lower per capita regional health expenditures with increased mortality rates [80, 81].

As demonstrated in our article, insights from SHAP plots may support clinical, managerial and people with lived experience intuition for risk classification, aid in creating more precise staging systems, and help identify risk inflection points for applications like suicide prediction. Finally, other newly developed explainability techniques may be a better option depending on the application [82–84].

Finally, it should be stressed that although the predictive models are working at the individual level, with distinct predictions on suicide risk made for each person, the approach is intended to study the effect of various factors of risk at the population and sub-population levels. The motivations for such an approach are two-fold. First, using such a predictive model for interventions at the individual level brings up various ethical and practical considerations, knowing that the level of errors at the individual predictions can be significant. Indeed, making proper use of individual suicide risk prediction in an operational setting would require better adjustments and safeguards to minimize possible harm, which is out of the scope of the current work. Second, we advocate that using individual predictions for evaluating the impact of some measures at the populational level provides better capabilities to evaluate suicide risk for some subgroups of the population (e.g., specific demographic or healthcare service subareas) and simulating the impact of some measures by altering some attributes of the individual (e.g., investment in mental healthcare, socio-economic deprivation).

## Limitation

There are several limitations of this study. First, the whole study was retrospectively conducted using INSPQ health administrative data for the prediction of suicide risk. The reliability of diagnoses and the fact that they are not mandatory in physician billing are the most important issues related to Quebec’s register [46]. It is first distributed similarly with the control group; secondly, many chronic diseases have been reasonably validated [46] and recent work validating with expected lifetime prevalence of schizophrenia in the Canadian Chronic Disease Surveillance System (circa 1% for Quebec and other populous provinces Canadian Chronic Disease Surveillance System (CCDSS) (canada.ca); and with ADHD long term outcomes similar with 1 or 2 more claims vs no claim [85]. Therefore, we know relatively little about suicide behaviors strongly associated with suicide deaths among the individuals in the control group. Second, this study was limited by the lack of information that may be relevant to suicide risk prediction, such as ethnicity, income, and education. Future studies should prioritize the collection and inclusion to overcome the limitation of information on relevant factors such as ethnicity, income, and education. Third, this study only covered suicides between 2002 and 2019, which could limit the applicability and generalizability of the study’s findings to the present time. Fourth, the study’s limited predictive value unanswered the question of why there is a difference in predictive value between both sexes. The final limitation of this study was the exclusion of medication data in the prediction risk of suicide since about 44% of patients had private insurance, and we could not access their medication use with the QICDSS. Future studies should explore the strategies for examining if medication data would enhance the model performance, as over 50% of the population are on the Quebec public drug plan.

## Conclusion

This study demonstrated the useful potential of explainable AI models as tools for decision-making and population-level suicide prevention actions. The ML models included individual, programmatic, systemic, and community levels variables available routinely to decision makers and planners in a public managed care system. Caution shall be exercised in the interpretation of variables associated in a predictive model since they are not causal, and other designs are required to establish the value of individual treatments. The next steps are to produce an intuitive user interface for decision makers, planners and other stakeholders like clinicians or representatives of families and people with live experience of suicidal behaviors or death by suicide. For example, how variations in the quality of local area primary care programs for depression or substance use disorders or increased in regional mental health and addiction budgets would lower suicide rates.

## Supporting information

S1 Table List of variables. (DOCX)

S2 Table Hyperparameters. (DOCX)

S1 Fig Reliability diagram before and after calibration. (DOCX)

## References

[1] Hopkins D, Rickwood DJ, Hallford DJ, Watsford C. Structured data vs. unstructured data in machine learning prediction models for suicidal behaviors: A systematic review and meta-analysis. Front Digit Health. 2022;132. doi: 10.3389/fdgth.2022.945006 35983407 PMC9378826

[2] Cerel J, Jordan JR, Duberstein PR. The impact of suicide on the family. Crisis: The Journal of Crisis Intervention and Suicide Prevention. 2008;29(1):38. doi: 10.1027/0227-5910.29.1.38 18389644

[3] Vasiliadis HM, Ngamini-Ngui A, Lesage A. Factors associated with suicide in the month following contact with different types of health services in Quebec. Psychiatric Services. 2015;66(2):121–6. doi: 10.1176/appi.ps.201400133 25270296

[4] Varin M, Orpana HM, Palladino E, Pollock NJ, Baker MM. Trends in Suicide Mortality in Canada by Sex and Age Group, 1981 to 2017: A Population-Based Time Series Analysis: Tendances de la mortalité par suicide au Canada selon le sexe et le groupe d’âge, 1981–2017: Une analyse de séries chronologiques dans la po. The Canadian Journal of Psychiatry. 2021;66(2):170–8. 32662296 10.1177/0706743720940565 PMC7917569

[5] Rassy J, Daneau D, Larue C, Rahme E, Low N, Lamarre S, et al. Measuring quality of care received by suicide attempters in the emergency department. Archives of suicide research. 2020;1–10. doi: 10.1080/13811118.2020.1793043 32715983

[6] Sanches A, Cardoso JMP, Delbem ACB. Identifying merge-beneficial software kernels for hardware implementation. In: Proceedings—2011 International Conference on Reconfigurable Computing and FPGAs, ReConFig 2011. 2011.

[7] Bernier S. La qualité des relations interpersonnelles chez les personnes âgées ayant le désir de mourir. Université du Québec à Trois-Rivières; 2019.

[8] Brådvik L. Suicide risk and mental disorders. Vol. 15, International journal of environmental research and public health. MDPI; 2018. p. 2028. 10.3390/ijerph15092028 PMC6165520 30227658

[9] Cavanagh JT, Carson AJ, Sharpe M, Lawrie SM, Li Z, Page A, et al. Attributable Risk of Psychiatric and Socioeconomic Risk Factors for Suicide from Individual-level, Population-based Studies: A Systematic Review. Psychol Med. 2003;33(3):395–405.12701661

[10] Fortin G, Ligier F, van Haaster I, Doyon C, Daneau D, Lesage A. Systematic Suicide Audit: An Enhanced Method to Assess System Gaps and Mobilize Leaders for Prevention. Quality Management in Healthcare. 2021;30(2):97–103. doi: 10.1097/QMH.0000000000000302 33633004

[11] Lesage A, Séguin M, Guy A, Daigle F, Bayle MN, Chawky N, et al. Systematic services audit of consecutive suicides in New Brunswick: the case for coordinating specialist mental health and addiction services. The Canadian Journal of Psychiatry. 2008;53(10):671–8. doi: 10.1177/070674370805301006 18940035

[12] Thibodeau L, Rahme E, Lachaud J, Pelletier É, Rochette L, John A, et al. Individual, programmatic and systemic indicators of the quality of mental health care using a large health administrative database: an avenue for preventing suicide mortality. Maladies Chroniques et Blessures au Canada. 2018;38. 10.24095/hpcdp.38.7/8.04 PMC6126560 30129717

[13] Ghahramani Z. Probabilistic machine learning and artificial intelligence. Nature. 2015;521(7553):452–9. doi: 10.1038/nature14541 26017444

[14] Abu-Raddad LJ, Chemaitelly H, Ayoub HH, al Kanaani Z, al Khal A, al Kuwari E, et al. Characterizing the Qatar advanced-phase SARS-CoV-2 epidemic. Sci Rep. 2021;11(1):1–15. 33737535 10.1038/s41598-021-85428-7 PMC7973743

[15] Shatte ABR, Hutchinson DM, Teague SJ. Machine learning in mental health: a scoping review of methods and applications. Psychol Med. 2019;49(9):1426–48. doi: 10.1017/S0033291719000151 30744717

[16] Jung JS, Park SJ, Kim EY, Na KS, Kim YJ, Kim KG. Prediction models for high risk of suicide in Korean adolescents using machine learning techniques. PLoS One. 2019. doi: 10.1371/journal.pone.0217639 31170212 PMC6553749

[17] Gradus JL, Rosellini AJ, Horváth-Puhó E, Street AE, Galatzer-Levy I, Jiang T, et al. Prediction of sex-specific suicide risk using machine learning and single-payer health care registry data from Denmark. JAMA Psychiatry. 2020;77(1):25–34. doi: 10.1001/jamapsychiatry.2019.2905 31642880 PMC6813578

[18] Kessler RC, Bossarte RM, Luedtke A, Zaslavsky AM, Zubizarreta JR. Suicide prediction models: a critical review of recent research with recommendations for the way forward. Mol Psychiatry. 2020;25(1):168–79. doi: 10.1038/s41380-019-0531-0 31570777 PMC7489362

[19] Walsh CG, Ribeiro JD, Franklin JC. Predicting suicide attempts in adolescents with longitudinal clinical data and machine learning. J Child Psychol Psychiatry. 2018. doi: 10.1111/jcpp.12916 29709069

[20] Qiu W, Chen H, Kaeberlein M, Lee SI. An explainable AI framework for interpretable biological age. medRxiv. 2022. 10.1016/S2666-7568(23)00189-7 37944549

[21] Adadi A, Berrada M. Peeking inside the black-box: a survey on explainable artificial intelligence (XAI). IEEE access. 2018;6:52138–60.

[22] Lundberg SM, Lee SI. A unified approach to interpreting model predictions. Adv Neural Inf Process Syst. 2017;30.

[23] Gholi Zadeh Kharrat F, Shydeo Brandão Miyoshi N, Cobre J, Mazzoncini De Azevedo-Marques J, Mazzoncini de Azevedo-Marques P, Cláudio Botazzo Delbem A. Feature sensitivity criterion-based sampling strategy from the Optimization based on Phylogram Analysis (Fs-OPA) and Cox regression applied to mental disorder datasets. PLoS One. 2020;15(7):e0235147. doi: 10.1371/journal.pone.0235147 32609749 PMC7329087

[24] Hall P, Gill N. An introduction to machine learning interpretability. O’Reilly Media, Incorporated; 2019.

[25] Kamath U, Liu J. Explainable Artificial Intelligence: An Introduction to Interpretable Machine Learning. Springer; 2021.

[26] Gunning D, Stefik M, Choi J, Miller T, Stumpf S, Yang GZ. XAI—Explainable artificial intelligence. Sci Robot. 2019;4(37):eaay7120. doi: 10.1126/scirobotics.aay7120 33137719

[27] Gunning D, Aha D. DARPA’s explainable artificial intelligence (XAI) program. AI Mag. 2019;40(2):44–58.

[28] Stiglic G, Kocbek P, Fijacko N, Zitnik M, Verbert K, Cilar L. Interpretability of machine learning‐based prediction models in healthcare. Wiley Interdiscip Rev Data Min Knowl Discov. 2020;10(5):e1379.

[29] Rothman D. Hands-On Explainable AI (XAI) with Python: Interpret, visualize, explain, and integrate reliable AI for fair, secure, and trustworthy AI apps. Packt Publishing Ltd; 2020.

[30] Barrigon ML, Cegla-Schvartzman F. Sex, gender, and suicidal behavior. Behavioral Neurobiology of Suicide and Self Harm. 2020;89–115. doi: 10.1007/7854_2020_165 32860593

[31] Freeman A, Mergl R, Kohls E, Székely A, Gusmao R, Arensman E, et al. A cross-national study on gender differences in suicide intent. BMC Psychiatry. 2017;17(1):1–11. 28662694 10.1186/s12888-017-1398-8 PMC5492308

[32] Canetto SS, Sakinofsky I. The gender paradox in suicide. Suicide Life Threat Behav. 1998;28(1):1–23. 9560163

[33] Hunt K, Sweeting H, Keoghan M, Platt S. Sex, gender role orientation, gender role attitudes and suicidal thoughts in three generations: A general population study. Soc Psychiatry Psychiatr Epidemiol. 2006;41:641–7. 16732400 10.1007/s00127-006-0074-y

[34] Parker G, Fletcher K, Hyett M, Hadzi-Pavlovic D, Barrett M, Synnott H. Measuring melancholia: the utility of a prototypic symptom approach. Psychol Med. 2009;39(6):989–98. doi: 10.1017/S0033291708004339 18796174

[35] Swami V, Stanistreet D, Payne S. Masculinities and suicide. Psychologist. 2008;21(4):308–11.

[36] Scourfield J. Suicidal masculinities. Sociol Res Online. 2005;10(2):35–44.

[37] Rutz W, Rihmer Z. Suicidality in men–practical issues, challenges, solutions. Journal of Men’s Health and Gender. 2007;4(4):393–401.

[38] Payne S, Swami V, Stanistreet DL. The social construction of gender and its influence on suicide: a review of the literature. J Mens Health. 2008;5(1):23–35.

[39] Webster Rudmin F, Ferrada‐Noli M, Skolbekken J. Questions of culture, age and gender in the epidemiology of suicide. Scand J Psychol. 2003;44(4):373–81. doi: 10.1111/1467-9450.00357 12887559

[40] Soole R, Kõlves K, De Leo D. Suicide in children: a systematic review. Archives of suicide research. 2015;19(3):285–304. doi: 10.1080/13811118.2014.996694 25517290

[41] Pelkonen M, Marttunen M. Child and adolescent suicide: epidemiology, risk factors, and approaches to prevention. Pediatric Drugs. 2003;5:243–65. doi: 10.2165/00128072-200305040-00004 12662120

[42] Brent D. Commentary: A time to reap and a time to sow: reducing the adolescent suicide rate now and in the future: commentary on Cha et al.(2018). Journal of child psychology and psychiatry. 2018;59(4):483–5. 29574731 10.1111/jcpp.12903

[43] Burns ER, Stevens JA, Lee R. The direct costs of fatal and non-fatal falls among older adults—United States. J Safety Res. 2016;58:99–103. doi: 10.1016/j.jsr.2016.05.001 27620939 PMC6823838

[44] Wang S, Ding X, Hu D, Zhang K, Huang D. A qualitative study on nurses’ reactions to inpatient suicide in a general hospital. Int J Nurs Sci. 2016;3(4):354–61.

[45] Schrijvers DL, Bollen J, Sabbe BGC. The gender paradox in suicidal behavior and its impact on the suicidal process. J Affect Disord. 2012;138(1–2):19–26. doi: 10.1016/j.jad.2011.03.050 21529962

[46] Blais C, Jean S, Sirois C, Rochette L, Plante C, Larocque I, et al. Quebec integrated chronic disease surveillance system (QICDSS), an innovative approach. Chronic Dis Inj Can. 2014;34(4). 25408182

[47] Pirkis J, Nicholas A, Gunnell D. The case for case–control studies in the field of suicide prevention. Epidemiol Psychiatr Sci. 2020;29:e62. 10.1017/S2045796019000581 PMC8061211 31571561

[48] Rubin DB. Missing data, imputation, and the bootstrap: Comment. J Am Stat Assoc. 1994;89(426):475–8.

[49] Dobbin KK, Simon RM. Optimally splitting cases for training and testing high dimensional classifiers. BMC Med Genomics. 2011;4(1):1–8. doi: 10.1186/1755-8794-4-31 21477282 PMC3090739

[50] Svetnik V, Liaw A, Tong C, Culberson JC, Sheridan RP, Feuston BP. Random forest: a classification and regression tool for compound classification and QSAR modeling. J Chem Inf Comput Sci. 2003;43(6):1947–58. doi: 10.1021/ci034160g 14632445

[51] Song S il Hong HT, Lee C Lee SB. A machine learning approach for predicting suicidal ideation in post stroke patients. Sci Rep. 2022;12(1):1–9. 36151132 10.1038/s41598-022-19828-8 PMC9508242

[52] Shahreen N, Subhani M, Rahman MM. Suicidal trend analysis of twitter using machine learning and neural network. In: 2018 international conference on Bangla speech and language processing (ICBSLP). IEEE; 2018. p. 1–5.

[53] Kaur P, Sharma M. Diagnosis of human psychological disorders using supervised learning and nature-inspired computing techniques: a meta-analysis. J Med Syst. 2019;43(7):1–30. doi: 10.1007/s10916-019-1341-2 31139933

[54] Abdellatif S, ben Hassine MA, ben Yahia S, Bouzeghoub A. ARCID: a new approach to deal with imbalanced datasets classification. In: International Conference on Current Trends in Theory and Practice of Informatics. Springer; 2018. p. 569–80.

[55] Mozafari AS, Gomes HS, Gagne C. Unsupervised Temperature Scaling: Robust Post-processing Calibration for Domain Shift. 2019.

[56] Laves MH, Ihler S, Kortmann KP, Ortmaier T. Well-calibrated model uncertainty with temperature scaling for dropout variational inference. arXiv preprint arXiv:190913550. 2019.

[57] Guo C, Pleiss G, Sun Y, Weinberger KQ. On calibration of modern neural networks. In: International Conference on Machine Learning. PMLR; 2017. p. 1321–30.

[58] Lundberg S. SHAP documentation. URL: https://shap readthedocs io (visited on 09/08/2021). 2021.

[59] Roth AE. Introduction to the Shapley value. The Shapley value. 1988;1–27.

[60] Ryu S, Lee H, Lee DK, Park K. Use of a machine learning algorithm to predict individuals with suicide ideation in the general population. Psychiatry Investig. 2018;15(11):1030. doi: 10.30773/pi.2018.08.27 30301301 PMC6258996

[61] Barros J, Morales S, Echávarri O, García A, Ortega J, Asahi T, et al. Suicide detection in Chile: proposing a predictive model for suicide risk in a clinical sample of patients with mood disorders. Brazilian Journal of Psychiatry. 2016;39:1–11. doi: 10.1590/1516-4446-2015-1877 27783715 PMC7112738

[62] Miché M, Studerus E, Meyer AH, Gloster AT, Beesdo-Baum K, Wittchen HU, et al. Prospective prediction of suicide attempts in community adolescents and young adults, using regression methods and machine learning. J Affect Disord. 2020;265:570–8. doi: 10.1016/j.jad.2019.11.093 31786028

[63] Passos IC, Mwangi B, Cao B, Hamilton JE, Wu MJ, Zhang XY, et al. Identifying a clinical signature of suicidality among patients with mood disorders: A pilot study using a machine learning approach. J Affect Disord. 2016;193:109–16. doi: 10.1016/j.jad.2015.12.066 26773901 PMC4744514

[64] Hettige NC, Nguyen TB, Yuan C, Rajakulendran T, Baddour J, Bhagwat N, et al. Classification of suicide attempters in schizophrenia using sociocultural and clinical features: A machine learning approach. Gen Hosp Psychiatry. 2017;47:20–8. doi: 10.1016/j.genhosppsych.2017.03.001 28807134

[65] Séguin M, Lesage A, Chawky N, Guy A, Daigle F, Girard G, et al. Suicide cases in New Brunswick from April 2002 to May 2003: the importance of better recognizing substance and mood disorder comorbidity. The Canadian Journal of Psychiatry. 2006;51(9):581–6. doi: 10.1177/070674370605100906 17007225

[66] Arsenault-Lapierre G, Kim C, Turecki G. Psychiatric diagnoses in 3275 suicides: a meta-analysis. BMC Psychiatry. 2004;4(1):1–11. doi: 10.1186/1471-244X-4-37 15527502 PMC534107

[67] Horvath A, Dras M, Lai CCW, Boag S. Predicting suicidal behavior without asking about suicidal ideation: machine learning and the role of borderline personality disorder criteria. Suicide Life Threat Behav. 2021;51(3):455–66. doi: 10.1111/sltb.12719 33185302

[68] Lesage A, Fortin G, Ligier F, Van Haaster I, Doyon C, Brouillard C, et al. Implementing a suicide audit in Montreal: taking suicide review further to make concrete recommendations for suicide prevention. Archives of suicide research. 2023;27(1):29–42. doi: 10.1080/13811118.2021.1965058 34470592

[69] Srinivasu PN, Sandhya N, Jhaveri RH, Raut R. From blackbox to explainable AI in healthcare: existing tools and case studies. Mobile Information Systems. 2022;2022.

[70] Merrick L, Taly A. The explanation game: Explaining machine learning models using shapley values. In: International Cross-Domain Conference for Machine Learning and Knowledge Extraction. Springer; 2020. p. 17–38.

[71] Thimoteo LM, Vellasco MM, do Amaral JM, Figueiredo K, Yokoyama CL, Marques E. Interpretable machine learning for COVID-19 diagnosis through clinical variables. In: Congresso Brasileiro de Automática-CBA. 2020.

[72] Leff HS, McPartland JC, Banks S, Dembling B, Fisher W, Allen IE. Service quality as measured by service fit and mortality among public mental health system service recipients. Ment Health Serv Res. 2004;6(2):93–107. doi: 10.1023/b:mhsr.0000024353.30425.ab 15224453

[73] Pirkola S, Sund R, Sailas E, Wahlbeck K. Community mental-health services and suicide rate in Finland: a nationwide small-area analysis. The Lancet. 2009;373(9658):147–53. doi: 10.1016/S0140-6736(08)61848-6 19097638

[74] Vasiliadis HM, Lesage A, Adair C, Boyer R. Service use for mental health reasons: cross-provincial differences in rates, determinants, and equity of access. The Canadian Journal of Psychiatry. 2005;50(10):614–9. doi: 10.1177/070674370505001007 16276852

[75] TANSELLA M, THORNICROFT G. A conceptual framework for mental health services: the matrix model. Psychol Med. 1998;28(3):503–8. doi: 10.1017/s0033291796005880 9626707

[76] Éthier S, Gagnon É, Couture M, Aubry F, Andrianova A, Smele S, et al. Démarche de mise en valeur des pratiques de bientraitance «ordinaire» en milieu d’hébergement au Québec: un travail de mobilisation de tous les acteurs concernés. Fonds de recherche Société et culture. https://frq. gouv. qc. ca/app/uploads …; 2021.

[77] Cavanagh JTO, Carson AJ, Sharpe M, Lawrie SM. Psychological autopsy studies of suicide: a systematic review. Psychol Med. 2003;33(3):395–405. doi: 10.1017/s0033291702006943 12701661

[78] Huỳnh C, Kisely S, Rochette L, Pelletier É, Morrison KB, Li S, et al. Measuring substance-related disorders using Canadian Administrative Health Databanks: interprovincial comparisons of recorded diagnostic rates, incidence proportions and mortality rate ratios. The Canadian Journal of Psychiatry. 2022;67(2):117–29. doi: 10.1177/07067437211043446 34569874 PMC8978214

[79] Tondo L, Albert MJ, Baldessarini RJ. Suicide rates in relation to health care access in the United States: an ecological study. Journal of Clinical Psychiatry. 2006;67(4):517–23. doi: 10.4088/jcp.v67n0402 16669716

[80] Claxton K, Martin S, Soares M, Rice N, Spackman E, Hinde S, et al. Methods for the estimation of the National Institute for Health and Care Excellence cost-effectiveness threshold. Health Technol Assess. 2015;19(14):1. doi: 10.3310/hta19140 25692211 PMC4781395

[81] Martin S, Longo F, Lomas J, Claxton K. Causal impact of social care, public health and healthcare expenditure on mortality in England: cross-sectional evidence for 2013/2014. BMJ Open. 2021;11(10):e046417. doi: 10.1136/bmjopen-2020-046417 34654700 PMC8559090

[82] Kovalev MS, Utkin L v, Kasimov EM. SurvLIME: A method for explaining machine learning survival models. Knowl Based Syst. 2020;203:106164.

[83] Lamy JB, Sekar B, Guezennec G, Bouaud J, Séroussi B. Explainable artificial intelligence for breast cancer: A visual case-based reasoning approach. Artif Intell Med. 2019;94:42–53. doi: 10.1016/j.artmed.2019.01.001 30871682

[84] Brown BW Jr, Hollander M, Korwar RM. Nonparametric tests of independence for censored data with application to heart transplant studies. FLORIDA STATE UNIV TALLAHASSEE DEPT OF STATISTICS; 1973.

[85] Diallo FB, Pelletier É, Vasiliadis H, Rochette L, Vincent A, Palardy S, et al. Morbidities and mortality of diagnosed attention deficit hyperactivity disorder (ADHD) over the youth lifespan: A population‐based retrospective cohort study. Int J Methods Psychiatr Res. 2022;31(1):e1903. doi: 10.1002/mpr.1903 34952999 PMC8886284

