# Supplementary material for: Explainable artificial intelligence models for predicting risk of suicide using health administrative data in Quebec
Source: PLoS One. 2024 Apr 3;19(4):e0301117. doi: 10.1371/journal.pone.0301117 (PMC10990247; doi:10.1371/journal.pone.0301117)
Supplement: S1 Table — (DOCX) [file pone.0301117.s001.docx]

**S 1 Tables: List of variables**

# Psychiatric Disorders Diagnosis

- Substance use disorder
  - Alcohol use disorder
  - drug use disorder
- Mood disorder
- Major depressive disorder
- Bipolar disorder
- Anxiety disorder
- Schizophrenia
- Personality disorders
- Other diagnosis
- ADHD

| derived variables | variable name |
| --- | --- |
| Substance use disorder | psydx_subuse_3 |
| Substance use disorder | psydx_subuse_6 |
| Substance use disorder | psydx_subuse_12 |
| Substance use disorder | psydx_subuse_24 |
| Substance use disorder | psydx_subuse_36 |
| Substance use disorder | psydx_subuse_48 |
| Substance use disorder | psydx_subuse_60 |
| Alcohol use disorder | Psydx_alcoholuse_3 |
| Alcohol use disorder | Psydx_alcoholuse_6 |
| Alcohol use disorder | Psydx_alcoholuse_12 |
| Alcohol use disorder | Psydx_alcoholuse_24 |
| Alcohol use disorder | Psydx_alcoholuse_36 |
| Alcohol use disorder | Psydx_alcoholuse_48 |
| Alcohol use disorder | Psydx_alcoholuse_60 |
| Drug use disorder | Psydx_druguse_3 |
| Drug use disorder | Psydx_druguse_6 |
| Drug use disorder | Psydx_druguse_12 |
| Drug use disorder | Psydx_druguse_24 |
| Drug use disorder | Psydx_druguse_36 |
| Drug use disorder | Psydx_druguse_48 |
| Drug use disorder | Psydx_druguse_60 |
| mood disorder | Psydx_mood_3 |
| mood disorder | Psydx_mood_6 |
| mood disorder | Psydx_mood_12 |
| mood disorder | Psydx_mood_24 |
| mood disorder | Psydx_mood_36 |
| mood disorder | Psydx_mood_48 |
| mood disorder | Psydx_mood_60 |
| Anxiety disorder | psydx_anx_3 |
| Anxiety disorder | psydx_anx_6 |
| Anxiety disorder | psydx_anx_12 |
| Anxiety disorder | psydx_anx_24 |
| Anxiety disorder | psydx_anx_36 |
| Anxiety disorder | psydx_anx_48 |
| Anxiety disorder | psydx_anx_60 |
| Major Depressive Disorder | Psydx_dep_3 |
| Major Depressive Disorder | Psydx_dep_6 |
| Major Depressive Disorder | Psydx_dep_12 |
| Major Depressive Disorder | Psydx_dep_24 |
| Major Depressive Disorder | Psydx_dep_36 |
| Major Depressive Disorder | Psydx_dep_48 |
| Major Depressive Disorder | Psydx_dep_60 |
| Bipolar disorder | Psydx_bipolar_3 |
| Bipolar disorder | Psydx_bipolar_6 |
| Bipolar disorder | Psydx_bipolar_12 |
| Bipolar disorder | Psydx_bipolar_24 |
| Bipolar disorder | Psydx_bipolar_36 |
| Bipolar disorder | Psydx_bipolar_48 |
| Bipolar disorder | Psydx_bipolar_60 |
| Schizophrenia | psydx_scz_3 |
| Schizophrenia | psydx_scz_6 |
| Schizophrenia | psydx_scz_12 |
| Schizophrenia | psydx_scz_24 |
| Schizophrenia | psydx_scz_36 |
| Schizophrenia | psydx_scz_48 |
| Schizophrenia | psydx_scz_60 |
| Personality disorder | psydx_pd_3 |
| Personality disorder | psydx_pd_6 |
| Personality disorder | psydx_pd_12 |
| Personality disorder | psydx_pd_24 |
| Personality disorder | psydx_pd_36 |
| Personality disorder | psydx_pd_48 |
| Personality disorder | psydx_pd_60 |
| ADHD | psydx_adhd_3 |
| ADHD | psydx_adhd_6 |
| ADHD | psydx_adhd_12 |
| ADHD | psydx_adhd_24 |
| ADHD | psydx_adhd_36 |
| ADHD | psydx_adhd_48 |
| ADHD | psydx_adhd_60 |
| Other diagnosis | psydx_otr_3 |
| Other diagnosis | psydx_otr_6 |
| Other diagnosis | psydx_otr_12 |
| Other diagnosis | psydx_otr_24 |
| Other diagnosis | psydx_otr_36 |
| Other diagnosis | psydx_otr_48 |
| Other diagnosis | psydx_otr_60 |

# Non-Pharmacological treatments for mental health disorders

- duration of hospitalisations for mental health reasons (continuous, sum of days)
- number of hospitalisations for mental health reasons (continuous)
- duration of hospitalisations for suicide attempt (continuous, sum of days)
- number of hospitalisations for suicide attempt (continuous)
- Number of care center visits for mental health reasons (continuous)
- number of general practitioner visits for mental health reasons (continuous)
- number of emergency room visits for mental health reasons (continuous)
- number of outpatient psychiatrist visits (continuous)
- number of other specialist visits for mental health reasons (continuous)
- number of psychotherapy visits with a psychiatrist (continuous)
- number of psychotherapy visits with a general practitioner (continuous)
- number of psychotherapy visits with another specialist (continuous)
- Number of outpatient paediatrician visits (continuous)
- No mental health services

| derived variables | variable name |
| --- | --- |
| Duration of hospit for suicide attempt (conti, # days) | duration_hosp_suicide_3 |
| duration of hospit for suicide attempt (conti, # days) | duration_hosp_suicide_6 |
| duration of hospit for suicide attempt (conti, # days) | duration_hosp_suicide_12 |
| duration of hospit for suicide attempt (conti, # days) | duration_hosp_suicide_24 |
| duration of hospit for suicide attempt (conti, # days) | duration_hosp_suicide_36 |
| duration of hospit for suicide attempt (conti, # days) | duration_hosp_suicide_48 |
| duration of hospit for suicide attempt (conti, # days) | duration_hosp_suicide_60 |
| # of hospit for suicide attempt (continuous) | #_hosp_suicide_3 |
| # of hospit for suicide attempt (continuous) | #_hosp_suicide_6 |
| # of hospit for suicide attempt (continuous) | #_hosp_suicide_12 |
| # of hospit for suicide attempt (continuous) | #_hosp_suicide_24 |
| # of hospit for suicide attempt (continuous) | #_hosp_suicide_36 |
| # of hospit for suicide attempt (continuous) | #_hosp_suicide_48 |
| # of hospit for suicide attempt (continuous) | #_hosp_suicide_60 |
| Duration of hospit for mh reasons (conti, # of days) | Duration_hosp_mh_3 |
| Duration of hospit for mh reasons (conti, # of days) | Duration_hosp_mh_6 |
| Duration of hospit for mh reasons (conti, # of days) | Duration_hosp_mh_12 |
| Duration of hospit for mh reasons (conti, # of days) | Duration_hosp_mh_24 |
| Duration of hospit for mh reasons (conti, # of days) | Duration_hosp_mh_36 |
| Duration of hospit for mh reasons (conti, # of days) | Duration_hosp_mh_48 |
| Duration of hospit for mh reasons (conti, # of days) | Duration_hosp_mh_60 |
| # of hospit for mh reasons (continuous) | #_hosp_mh_3 |
| # of hospit for mh reasons (continuous) | #_hosp_mh_6 |
| # of hospit for mh reasons (continuous) | #_hosp_mh_12 |
| # of hospit for mh reasons (continuous) | #_hosp_mh_24 |
| # of hospit for mh reasons (continuous) | #_hosp_mh_36 |
| # of hospit for mh reasons (continuous) | #_hosp_mh_48 |
| # of hospit for mh reasons (continuous) | #_hosp_mh_60 |
| # of outpatient paediatrician visits (continuous) | #_outpat_pediatrician_3 |
| # of outpatient paediatrician visits (continuous) | #_outpat_pediatrician_6 |
| # of outpatient paediatrician visits (continuous) | #_outpat_pediatrician_12 |
| # of outpatient paediatrician visits (continuous) | #_outpat_pediatrician_24 |
| # of outpatient paediatrician visits (continuous) | #_outpat_pediatrician_36 |
| # of outpatient paediatrician visits (continuous) | #_outpat_pediatrician_48 |
| # of outpatient paediatrician visits (continuous) | #_outpat_pediatrician_60 |
| Number of Care center for mental health reasons (continuous) | #_Carectr_mh_3 |
| Number of Care center for mental health reasons (continuous) | #_Carectr_mh_6 |
| Number of Care center for mental health reasons (continuous) | #_Carectr_mh_12 |
| Number of Care center for mental health reasons (continuous) | #_Carectr_mh_24 |
| Number of Care center for mental health reasons (continuous) | #_Carectr_mh_36 |
| Number of Care center for mental health reasons (continuous) | #_Carectr_mh_48 |
| Number of Care center for mental health reasons (continuous) | #_Carectr_mh_60 |
| # of emergency visits for mh reasons (continuous) | #_ER_mh_3 |
| # of emergency visits for mh reasons (continuous) | #_ER_mh_6 |
| # of emergency visits for mh reasons (continuous) | #_ER_mh_12 |
| # of emergency visits for mh reasons (continuous) | #_ER_mh_24 |
| # of emergency visits for mh reasons (continuous) | #_ER_mh_36 |
| # of emergency visits for mh reasons (continuous) | #_ER_mh_48 |
| # of emergency visits for mh reasons (continuous) | #_ER_mh_60 |
| # of GP visits for mh reasons (continuous) | #_gp_mh_3 |
| # of GP visits for mh reasons (continuous) | #_gp_mh_6 |
| # of GP visits for mh reasons (continuous) | #_gp_mh_12 |
| # of GP visits for mh reasons (continuous) | #_gp_mh_24 |
| # of GP visits for mh reasons (continuous) | #_gp_mh_36 |
| # of GP visits for mh reasons (continuous) | #_gp_mh_48 |
| # of GP visits for mh reasons (continuous) | #_gp_mh_60 |
| # of outpatient psychiatrist visits (continuous) | #_psy_mh_3 |
| # of outpatient psychiatrist visits (continuous) | #_psy_mh_6 |
| # of outpatient psychiatrist visits (continuous) | #_psy_mh_12 |
| # of outpatient psychiatrist visits (continuous) | #_psy_mh_24 |
| # of outpatient psychiatrist visits (continuous) | #_psy_mh_36 |
| # of outpatient psychiatrist visits (continuous) | #_psy_mh_48 |
| # of outpatient psychiatrist visits (continuous) | #_psy_mh_60 |
| # of psychotherapy visits with a psychiatrist (conti) | #_psychotx_psy_3 |
| # of psychotherapy visits with a psychiatrist (conti) | #_psychotx_psy_6 |
| # of psychotherapy visits with a psychiatrist (conti) | #_psychotx_psy_12 |
| # of psychotherapy visits with a psychiatrist (conti) | #_psychotx_psy_24 |
| # of psychotherapy visits with a psychiatrist (conti) | #_psychotx_psy_36 |
| # of psychotherapy visits with a psychiatrist (conti) | #_psychotx_psy_48 |
| # of psychotherapy visits with a psychiatrist (conti) | #_psychotx_psy_60 |
| # of other specialist visits for mh reasons (conti) | #_spc_mh_3 |
| # of other specialist visits for mh reasons (conti) | #_spc_mh_6 |
| # of other specialist visits for mh reasons (conti) | #_spc_mh_12 |
| # of other specialist visits for mh reasons (conti) | #_spc_mh_24 |
| # of other specialist visits for mh reasons (conti) | #_spc_mh_36 |
| # of other specialist visits for mh reasons (conti) | #_spc_mh_48 |
| # of other specialist visits for mh reasons (conti) | #_spc_mh_60 |
| # of psychotherapy visits with a GP (conti) | #_psychotx_gp_3 |
| # of psychotherapy visits with a GP (conti) | #_psychotx_gp_6 |
| # of psychotherapy visits with a GP (conti) | #_psychotx_gp_12 |
| # of psychotherapy visits with a GP (conti) | #_psychotx_gp_24 |
| # of psychotherapy visits with a GP (conti) | #_psychotx_gp_36 |
| # of psychotherapy visits with a GP (conti) | #_psychotx_gp_48 |
| # of psychotherapy visits with a GP (conti) | #_psychotx_gp_60 |
| # of psychotherapy visits with other specialist (conti) | #_psychotx_other_3 |
| # of psychotherapy visits with other specialist (conti) | #_psychotx_other_6 |
| # of psychotherapy visits with other specialist (conti) | #_psychotx_other_12 |
| # of psychotherapy visits with other specialist (conti) | #_psychotx_other_24 |
| # of psychotherapy visits with other specialist (conti) | #_psychotx_other_36 |
| # of psychotherapy visits with other specialist (conti) | #_psychotx_other_48 |
| # of psychotherapy visits with other specialist (conti) | #_psychotx_other_60 |
| No mental health services | No_mh_services_3 |
| No mental health services | No_mh_services_6 |
| No mental health services | No_mh_services_12 |
| No mental health services | No_mh_services_24 |
| No mental health services | No_mh_services_36 |
| No mental health services | No_mh_services_48 |
| No mental health services | No_mh_services_60 |

# Physical diagnosis

- Dementia
- Neurological disease
- Endocrine system disorder
- Trauma
- Respiratory disorder
- Infectious disease
- Digestive disorder
- Cardiovascular disorder
- Cancer
- Other physical disorder
- Charlson/elixhauser index with psy (continuous)
- Charlson/elixhauser index without psy (continuous)

| derived variables | variable name |
| --- | --- |
| dementia | physdx_dem_3 |
| dementia | physdx _dem_6 |
| dementia | physdx _dem_12 |
| dementia | physdx _dem_24 |
| dementia | physdx _dem_36 |
| dementia | physdx _dem_48 |
| dementia | physdx _dem_60 |
| neurological disease | physdx _neuro_3 |
| neurological disease | physdx _neuro_6 |
| neurological disease | physdx _neuro_12 |
| neurological disease | physdx _neuro_24 |
| neurological disease | physdx _neuro_36 |
| neurological disease | physdx _neuro_48 |
| neurological disease | physdx _neuro_60 |
| endocrine system disorder | physdx _endo_3 |
| endocrine system disorder | physdx _endo_6 |
| endocrine system disorder | physdx _endo_12 |
| endocrine system disorder | physdx _endo_24 |
| endocrine system disorder | physdx _endo_36 |
| endocrine system disorder | physdx _endo_48 |
| endocrine system disorder | physdx _endo_60 |
| trauma | physdx _trauma_3 |
| trauma | physdx _trauma_6 |
| trauma | physdx _trauma_12 |
| trauma | physdx _trauma_24 |
| trauma | physdx _trauma_36 |
| trauma | physdx _trauma_48 |
| trauma | physdx _trauma_60 |
| respiratory disorder | physdx _resp_3 |
| respiratory disorder | physdx _resp_6 |
| respiratory disorder | physdx _resp_12 |
| respiratory disorder | physdx _resp_24 |
| respiratory disorder | physdx _resp_36 |
| respiratory disorder | physdx _resp_48 |
| respiratory disorder | physdx _resp_60 |
| infectious disease | physdx _infec_3 |
| infectious disease | physdx _infec_6 |
| infectious disease | physdx _infec_12 |
| infectious disease | physdx _infec_24 |
| infectious disease | physdx _infec_36 |
| infectious disease | physdx _infec_48 |
| infectious disease | physdx _infec_60 |
| digestive disorder | physdx _diges_3 |
| digestive disorder | physdx _diges_6 |
| digestive disorder | physdx _diges_12 |
| digestive disorder | physdx _diges_24 |
| digestive disorder | physdx _diges_36 |
| digestive disorder | physdx _diges_48 |
| digestive disorder | physdx _diges_60 |
| cardiovascular disorder | physdx _cvd_3 |
| cardiovascular disorder | physdx _cvd_6 |
| cardiovascular disorder | physdx _cvd_12 |
| cardiovascular disorder | physdx _cvd_24 |
| cardiovascular disorder | physdx _cvd_36 |
| cardiovascular disorder | physdx _cvd_48 |
| cardiovascular disorder | physdx _cvd_60 |
| cancer | physdx _cncr_3 |
| cancer | physdx _cncr_6 |
| cancer | physdx _cncr_12 |
| cancer | physdx _cncr_24 |
| cancer | physdx _cncr_36 |
| cancer | physdx _cncr_48 |
| cancer | physdx _cncr_60 |
| other physical disorders | physdx _otr_3 |
| other physical disorders | physdx _otr_6 |
| other physical disorders | physdx _otr_12 |
| other physical disorders | physdx _otr_24 |
| other physical disorders | physdx _otr_36 |
| other physical disorders | physdx _otr_48 |
| other physical disorders | physdx _otr_60 |
| charlson/elixhauser index with psy (conti) | physdx_comorbidity_withpsy_3 |
| charlson/elixhauser index with psy (conti) | physdx_comorbid_withpsy_6 |
| charlson/elixhauser index with psy (conti) | physdx_comorbidity_withpsy_12 |
| charlson/elixhauser index with psy (conti) | physdx_comorbidity_withpsy_24 |
| charlson/elixhauser index with psy (conti) | physdx_comorbidity_withpsy_36 |
| charlson/elixhauser index with psy (conti) | physdx_comorbidity_withpsy_48 |
| charlson/elixhauser index with psy (conti) | physdx_comorbidity_withpsy_60 |
| charlson/elixhauser index without psy (conti) | physdx_comorbidity_withoutpsy_3 |
| charlson/elixhauser index without psy (conti) | physdx_comorbid_withoutpsy_6 |
| charlson/elixhauser index without psy (conti) | physdx_comorbidity_withoutpsy_12 |
| charlson/elixhauser index without psy (conti) | physdx_comorbidity_withoutpsy_24 |
| charlson/elixhauser index without psy (conti) | physdx_comorbidity_withoutpsy_36 |
| charlson/elixhauser index without psy (conti) | physdx_comorbidity_withoutpsy_48 |
| charlson/elixhauser index without psy (conti) | physdx_comorbidity_withoutpsy_60 |

# Non-pharmacological treatments for physical health disorders

- duration of hospitalisations for physical health reasons (continuous, sum of days)
- number of hospitalisations for physical health reasons (continuous)
- care center visits/plays for physical health reasons*
- number of general practitioner visits for physical reasons (continuous)*
- number of emergency room visits for physical reasons (continuous)*
- number of outpatient specialist visits for physical health reasons (continuous)*
- number of outpatient paediatrician visits (continuous)*

| derived variables | variable name |
| --- | --- |
| Duration of hospit for phys reasons (conti, # of days) | Duration_hosp_phys_3 |
| Duration of hospit for phys reasons (conti, # of days) | Duration_hosp_phys_6 |
| Duration of hospit for phys reasons (conti, # of days) | Duration_hosp_phys_12 |
| Duration of hospit for phys reasons (conti, # of days) | Duration_hosp_phys_24 |
| Duration of hospit for phys reasons (conti, # of days) | Duration_hosp_phys_36 |
| Duration of hospit for phys reasons (conti, # of days) | Duration_hosp_phys_48 |
| Duration of hospit for phys reasons (conti, # of days) | Duration_hosp_phys_60 |
| # of hospit for phys reasons (continuous) | #_hosp_phys_3 |
| # of hospit for phys reasons (continuous) | #_hosp_phys_6 |
| # of hospit for phys reasons (continuous) | #_hosp_phys_12 |
| # of hospit for phys reasons (continuous) | #_hosp_phys_24 |
| # of hospit for phys reasons (continuous) | #_hosp_phys_36 |
| # of hospit for phys reasons (continuous) | #_hosp_phys_48 |
| # of hospit for phys reasons (continuous) | #_hosp_phys_60 |
| Care center for physical health reasons | Carectr_phys_3 |
| Care center for physical health reasons | Carectr_phys_6 |
| Care center for physical health reasons | Carectr_phys_12 |
| Care center for physical health reasons | Carectr_phys_24 |
| Care center for physical health reasons | Carectr_phys_36 |
| Care center for physical health reasons | Carectr_phys_48 |
| Care center for physical health reasons | Carectr_phys_60 |
| # of outpatient specialist visit for phys reasons (cont) | #_spc_phys_3 |
| # of outpatient specialist visit for phys reasons (cont) | #_spc_phys_6 |
| # of outpatient specialist visit for phys reasons (cont) | #_spc_phys_12 |
| # of outpatient specialist visit for phys reasons (cont) | #_spc_phys_24 |
| # of outpatient specialist visit for phys reasons (cont) | #_spc_phys_36 |
| # of outpatient specialist visit for phys reasons (cont) | #_spc_phys_48 |
| # of outpatient specialist visit for phys reasons (cont) | #_spc_phys_60 |
| # of GP visits for phys reasons (continuous) | #_gp_phys_3 |
| # of GP visits for phys reasons (continuous) | #_gp_phys_6 |
| # of GP visits for phys reasons (continuous) | #_gp_phys_12 |
| # of GP visits for phys reasons (continuous) | #_gp_phys_24 |
| # of GP visits for phys reasons (continuous) | #_gp_phys_36 |
| # of GP visits for phys reasons (continuous) | #_gp_phys_48 |
| # of GP visits for phys reasons (continuous) | #_gp_phys_60 |
| # of emergency visits for phys reasons (continuous) | #_ER_phys_3 |
| # of emergency visits for phys reasons (continuous) | #_ER_phys_6 |
| # of emergency visits for phys reasons (continuous) | #_ER_phys_12 |
| # of emergency visits for phys reasons (continuous) | #_ER_phys_24 |
| # of emergency visits for phys reasons (continuous) | #_ER_phys_36 |
| # of emergency visits for phys reasons (continuous) | #_ER_phys_48 |
| # of emergency visits for phys reasons (continuous) | #_ER_phys_60 |

## Individual Socio-Demographic Variables

- Age (continuous)
- age group: 15-24
- age group: 25-34
- age group: 35-44
- age group: 45-54
- age group: 55-64
- age group: 65-74
- age group: 75-84
- age group: ≥85
- Sex
- Location – rural
- Location – non-rural
- Location – missing data
- rss 01 bas saint-laurent
- rss 02 saguenay-lac-saint-jean
- rss 03 capitale-nationale
- rss 04 mauricie et centre-du-québec
- rss 05 estrie
- rss 06 montréal
- rss 07 outaouais
- rss 08 abitibi-témiscamingue
- rss 09 côte-nord
- rss 10 nord-du-québec
- rss 11gaspésie-îles-de-la-madeleine
- rss 12 chaudière-appalaches
- rss 13 laval
- rss 14 lanaudière
- rss 15 laurentides
- rss 16 montérégie
- Adherence to the public drug plan (RAMQ) (dichotomous)

| derived variables | variable name |
| --- | --- |
| Age (continuous) | Age_continuous |
| age group: 15-24 | age_15-24 |
| age group: 25-34 | age_25-34 |
| age group: 35-44 | age_35-44 |
| age group: 45-54 | age_45-54 |
| age group: 55-64 | age_55-64 |
| age group: 65-74 | age_65-74 |
| age group: 75-84 | age_75-84 |
| age group: ≥85 | age_85+ |
| sex male | sex_m |
| sex female | sex_f |
| rss 01 bas-saint-laurent | loc_rss_01_3 |
| rss 01 bas-saint-laurent | loc_rss_01_6 |
| rss 01 bas-saint-laurent | loc_rss_01_12 |
| rss 01 bas-saint-laurent | loc_rss_01_24 |
| rss 01 bas-saint-laurent | loc_rss_01_36 |
| rss 01 bas-saint-laurent | loc_rss_01_48 |
| rss 01 bas-saint-laurent | loc_rss_01_60 |
| rss 02 saguenay-lac-saint-jean | loc_rss_02_3 |
| rss 02 saguenay-lac-saint-jean | loc_rss_02_6 |
| rss 02 saguenay-lac-saint-jean | loc_rss_02_12 |
| rss 02 saguenay-lac-saint-jean | loc_rss_02_24 |
| rss 02 saguenay-lac-saint-jean | loc_rss_02_36 |
| rss 02 saguenay-lac-saint-jean | loc_rss_02_48 |
| rss 02 saguenay-lac-saint-jean | loc_rss_02_60 |
| rss 03 capitale-nationale | loc_rss_03_3 |
| rss 03 capitale-nationale | loc_rss_03_6 |
| rss 03 capitale-nationale | loc_rss_03_12 |
| rss 03 capitale-nationale | loc_rss_03_24 |
| rss 03 capitale-nationale | loc_rss_03_36 |
| rss 03 capitale-nationale | loc_rss_03_48 |
| rss 03 capitale-nationale | loc_rss_03_60 |
| rss 04 mauricie et centre-du-québec | loc_rss_04_3 |
| rss 04 mauricie et centre-du-québec | loc_rss_04_6 |
| rss 04 mauricie et centre-du-québec | loc_rss_04_12 |
| rss 04 mauricie et centre-du-québec | loc_rss_04_24 |
| rss 04 mauricie et centre-du-québec | loc_rss_04_36 |
| rss 04 mauricie et centre-du-québec | loc_rss_04_48 |
| rss 04 mauricie et centre-du-québec | loc_rss_04_60 |
| rss 05 estrie | loc_rss_05_3 |
| rss 05 estrie | loc_rss_05_6 |
| rss 05 estrie | loc_rss_05_12 |
| rss 05 estrie | loc_rss_05_24 |
| rss 05 estrie | loc_rss_05_36 |
| rss 05 estrie | loc_rss_05_48 |
| rss 05 estrie | loc_rss_05_60 |
| rss 06 montréal | loc_rss_06_3 |
| rss 06 montréal | loc_rss_06_6 |
| rss 06 montréal | loc_rss_06_12 |
| rss 06 montréal | loc_rss_06_24 |
| rss 06 montréal | loc_rss_06_36 |
| rss 06 montréal | loc_rss_06_48 |
| rss 06 montréal | loc_rss_06_60 |
| rss 07 outaouais | loc_rss_07_3 |
| rss 07 outaouais | loc_rss_07_6 |
| rss 07 outaouais | loc_rss_07_12 |
| rss 07 outaouais | loc_rss_07_24 |
| rss 07 outaouais | loc_rss_07_36 |
| rss 07 outaouais | loc_rss_07_48 |
| rss 07 outaouais | loc_rss_07_60 |
| 08 abitibi-témiscamingue | loc_rss_08_3 |
| rss 08 abitibi-témiscamingue | loc_rss_08_6 |
| rss 08 abitibi-témiscamingue | loc_rss_08_12 |
| rss 08 abitibi-témiscamingue | loc_rss_08_24 |
| 08 abitibi-témiscamingue | loc_rss_08_36 |
| rss 08 abitibi-témiscamingue | loc_rss_08_48 |
| rss 08 abitibi-témiscamingue | loc_rss_08_60 |
| rss 09 côte-nord | loc_rss_09_3 |
| rss 09 côte-nord | loc_rss_09_6 |
| rss 09 côte-nord | loc_rss_09_12 |
| rss 09 côte-nord | loc_rss_09_24 |
| rss 09 côte-nord | loc_rss_09_36 |
| rss 09 côte-nord | loc_rss_09_48 |
| rss 09 côte-nord | loc_rss_09_60 |
| rss 10 nord-du-québec | loc_rss_10_3 |
| rss 10 nord-du-québec | loc_rss_10_6 |
| rss 10 nord-du-québec | loc_rss_10_12 |
| rss 10 nord-du-québec | loc_rss_10_24 |
| rss 10 nord-du-québec | loc_rss_10_36 |
| rss 10 nord-du-québec | loc_rss_10_48 |
| rss 10 nord-du-québec | loc_rss_10_60 |
| rss 11 gaspésie-îles-de-la-madeleine | loc_rss_11_3 |
| rss 11 gaspésie-îles-de-la-madeleine | loc_rss_11_6 |
| rss 11 gaspésie-îles-de-la-madeleine | loc_rss_11_12 |
| rss 11 gaspésie-îles-de-la-madeleine | loc_rss_11_24 |
| rss 11 gaspésie-îles-de-la-madeleine | loc_rss_11_36 |
| rss 11 gaspésie-îles-de-la-madeleine | loc_rss_11_48 |
| rss 11 gaspésie-îles-de-la-madeleine | loc_rss_11_60 |
| rss 12 chaudière-appalaches | loc_rss_12_3 |
| rss 12 chaudière-appalaches | loc_rss_12_6 |
| rss 12 chaudière-appalaches | loc_rss_12_12 |
| rss 12 chaudière-appalaches | loc_rss_12_24 |
| rss 12 chaudière-appalaches | loc_rss_12_36 |
| rss 12 chaudière-appalaches | loc_rss_12_48 |
| rss 12 chaudière-appalaches | loc_rss_12_60 |
| rss 13 laval | loc_rss_13_3 |
| rss 13 laval | loc_rss_13_6 |
| rss 13 laval | loc_rss_13_12 |
| rss 13 laval | loc_rss_13_24 |
| rss 13 laval | loc_rss_13_36 |
| rss 13 laval | loc_rss_13_48 |
| rss 13 laval | loc_rss_13_60 |
| rss 14 lanaudière | loc_rss_14_3 |
| rss 14 lanaudière | loc_rss_14_6 |
| rss 14 lanaudière | loc_rss_14_12 |
| rss 14 lanaudière | loc_rss_14_24 |
| rss 14 lanaudière | loc_rss_14_36 |
| rss 14 lanaudière | loc_rss_14_48 |
| rss 14 lanaudière | loc_rss_14_60 |
| rss 15 laurentides | loc_rss_15_3 |
| rss 15 laurentides | loc_rss_15_6 |
| rss 15 laurentides | loc_rss_15_12 |
| rss 15 laurentides | loc_rss_15_24 |
| rss 15 laurentides | loc_rss_15_36 |
| rss 15 laurentides | loc_rss_15_48 |
| rss 15 laurentides | loc_rss_15_60 |
| rss 16 montérégie | loc_rss_16_3 |
| rss 16 montérégie | loc_rss_16_6 |
| rss 16 montérégie | loc_rss_16_12 |
| rss 16 montérégie | loc_rss_16_24 |
| rss 16 montérégie | loc_rss_16_36 |
| rss 16 montérégie | loc_rss_16_48 |
| rss 16 montérégie | loc_rss_16_60 |
| location nonrural | nonrural_3 |
| location nonrural | nonrural_6 |
| location nonrural | nonrural_12 |
| location nonrural | nonrural_24 |
| location nonrural | nonrural_36 |
| location nonrural | nonrural_48 |
| location nonrural | nonrural_60 |
| location rural | rural_3 |
| location rural | rural_6 |
| location rural | rural_12 |
| location rural | rural_24 |
| location rural | rural_36 |
| location rural | rural_48 |
| location rural | rural_60 |
| location missing data | loc_missing_3 |
| location missing data | loc_missing_6 |
| location missing data | loc_missing_12 |
| location missing data | loc_missing_24 |
| location missing data | loc_missing_36 |
| location missing data | loc_missing_48 |
| location missing data | loc_missing_60 |

# Environmental Variables

## Deprivation Index

- Material deprivation (from 1, least deprived to 5, most deprived)
- Social deprivation (from 1, least deprived to 5, most deprived)

| derived variables | variable name |
| --- | --- |
| material deprivation (1-5) | matdep_3 |
| material deprivation (1-5) | matdep_6 |
| material deprivation (1-5) | matdep_12 |
| material deprivation (1-5) | matdep_24 |
| material deprivation (1-5) | matdep_36 |
| material deprivation (1-5) | matdep_48 |
| material deprivation (1-5) | matdep_60 |
| social deprivation (1-5) | socdep_3 |
| social deprivation (1-5) | socdep_6 |
| social deprivation (1-5) | socdep_12 |
| social deprivation (1-5) | socdep_24 |
| social deprivation (1-5) | socdep_36 |
| social deprivation (1-5) | socdep_48 |
| social deprivation (1-5) | socdep_60 |

# System variables

## Health System Environment (Health System)

- Mental health budget
- Bas-Saint-Laurent
- Saguenay-Lac-Saint-Jean
- Capitale-Nationale
- Mauricie et Centre-du-Québec
- Estrie
- Montréal
- Outaouais
- Abitibi-Témiscamingue
- Côte-Nord
- Nord-du-Québec
- Gaspésie-îles-de-la-Madeleine
- Chaudière-Appalaches
- Laval
- Lanaudière
- Laurentides
- Montérégie
- Addictions budget
- Bas-Saint-Laurent
- Saguenay-Lac-Saint-Jean
- Capitale-Nationale
- Mauricie et Centre-du-Québec
- Estrie
- Montréal
- Outaouais
- Abitibi-Témiscamingue
- Côte-Nord
- Nord-du-Québec
- Gaspésie-îles-de-la-Madeleine
- Chaudière-Appalaches
- Laval
- Lanaudière
- Laurentides
- Montérégie
- regional mental health budget ($/capita)
- 2018-2019
- 2017-2018
- 2016-2017
- 2015-2016
- regional addictions health budget ($/capita)
- 2018-2019
- 2017-2018
- 2016-2017
- 2015-2016

| derived variables | variable name |
| --- | --- |
| rss 01 bas-saint-laurent mental health budget | rss_01_mh_3 |
| rss 01 bas-saint-laurent mental health budget | rss_01_mh_6 |
| rss 01 bas-saint-laurent mental health budget | rss_01_mh_12 |
| rss 01 bas-saint-laurent mental health budget | rss_01_mh_24 |
| rss 01 bas-saint-laurent mental health budget | rss_01_mh_36 |
| rss 01 bas-saint-laurent mental health budget | rss_01_mh_48 |
| rss 01 bas-saint-laurent mental health budget | rss_01_mh_60 |
| rss 02 saguenay-lac-saint-jean mental health budget | rss_02_mh_3 |
| rss 02 saguenay-lac-saint-jean mental health budget | rss_02_mh_6 |
| rss 02 saguenay-lac-saint-jean mental health budget | rss_02_mh_12 |
| rss 02 saguenay-lac-saint-jean mental health budget | rss_02_mh_24 |
| rss 02 saguenay-lac-saint-jean mental health budget | rss_02_mh_36 |
| rss 02 saguenay-lac-saint-jean mental health budget | rss_02_mh_48 |
| rss 02 saguenay-lac-saint-jean mental health budget | rss_02_mh_60 |
| rss 03 capitale-nationale mental health budget | rss_03_mh_3 |
| rss 03 capitale-nationale mental health budget | rss_03_mh_6 |
| rss 03 capitale-nationale mental health budget | rss_03_mh_12 |
| rss 03 capitale-nationale mental health budget | rss_03_mh_14 |
| rss 03 capitale-nationale mental health budget | rss_03_mh_36 |
| rss 03 capitale-nationale mental health budget | rss_03_mh_48 |
| rss 03 capitale-nationale mental health budget | rss_03_mh_60 |
| rss 04 mauricie et centre-du-québec mental health budget | rss_04_mh_3 |
| rss 04 mauricie et centre-du-québec mental health budget | rss_04_mh_6 |
| rss 04 mauricie et centre-du-québec mental health budget | rss_04_mh_12 |
| rss 04 mauricie et centre-du-québec mental health budget | rss_04_mh_24 |
| rss 04 mauricie et centre-du-québec mental health budget | rss_04_mh_36 |
| rss 04 mauricie et centre-du-québec mental health budget | rss_04_mh_48 |
| rss 04 mauricie et centre-du-québec mental health budget | rss_04_mh_60 |
| rss 05 estrie mental health budget | rss_05_mh_3 |
| rss 05 estrie mental health budget | rss_05_mh_6 |
| rss 05 estrie mental health budget | rss_05_mh_12 |
| rss 05 estrie mental health budget | rss_05_mh_24 |
| rss 05 estrie mental health budget | rss_05_mh_36 |
| rss 05 estrie mental health budget | rss_05_mh_48 |
| rss 05 estrie mental health budget | rss_05_mh_60 |
| rss 06 montréal mental health budget | rss_06_mh_3 |
| rss 06 montréal mental health budget | rss_06_mh_6 |
| rss 06 montréal mental health budget | rss_06_mh_12 |
| rss 06 montréal mental health budget | rss_06_mh_24 |
| rss 06 montréal mental health budget | rss_06_mh_36 |
| rss 06 montréal mental health budget | rss_06_mh_48 |
| rss 06 montréal mental health budget | rss_06_mh_60 |
| rss 07 outaouais mental health budget | rss_07_mh_3 |
| rss 07 outaouais mental health budget | rss_07_mh_6 |
| rss 07 outaouais mental health budget | rss_07_mh_12 |
| rss 07 outaouais mental health budget | rss_07_mh_24 |
| rss 07 outaouais mental health budget | rss_07_mh_36 |
| rss 07 outaouais mental health budget | rss_07_mh_48 |
| rss 07 outaouais mental health budget | rss_07_mh_60 |
| rss 08 abitibi-témiscamingue mental health budget | rss_08_mh_3 |
| rss 08 abitibi-témiscamingue mental health budget | rss_08_mh_6 |
| rss 08 abitibi-témiscamingue mental health budget | rss_08_mh_12 |
| rss 08 abitibi-témiscamingue mental health budget | rss_08_mh_24 |
| rss 08 abitibi-témiscamingue mental health budget | rss_08_mh_36 |
| rss 08 abitibi-témiscamingue mental health budget | rss_08_mh_48 |
| rss 08 abitibi-témiscamingue mental health budget | rss_08_mh_60 |
| rss 09 côte-nord mental health budget | rss_09_mh_3 |
| rss 09 côte-nord mental health budget | rss_09_mh_6 |
| rss 09 côte-nord mental health budget | rss_09_mh_12 |
| rss 09 côte-nord mental health budget | rss_09_mh_24 |
| rss 09 côte-nord mental health budget | rss_09_mh_36 |
| rss 09 côte-nord mental health budget | rss_09_mh_48 |
| rss 09 côte-nord mental health budget | rss_09_mh_60 |
| rss 10 nord-du-québec mental health budget | rss_10_mh_3 |
| rss 10 nord-du-québec mental health budget | rss_10_mh_6 |
| rss 10 nord-du-québec mental health budget | rss_10_mh_12 |
| rss 10 nord-du-québec mental health budget | rss_10_mh_24 |
| rss 10 nord-du-québec mental health budget | rss_10_mh_36 |
| rss 10 nord-du-québec mental health budget | rss_10_mh_48 |
| rss 10 nord-du-québec mental health budget | rss_10_mh_60 |
| rss 11 gaspésie-îles-de-la-madeleine mental health budget | rss_11_mh_3 |
| rss 11 gaspésie-îles-de-la-madeleine mental health budget | rss_11_mh_6 |
| rss 11 gaspésie-îles-de-la-madeleine mental health budget | rss_11_mh_12 |
| rss 11 gaspésie-îles-de-la-madeleine mental health budget | rss_11_mh_24 |
| rss 11 gaspésie-îles-de-la-madeleine mental health budget | rss_11_mh_36 |
| rss 11 gaspésie-îles-de-la-madeleine mental health budget | rss_11_mh_48 |
| rss 11 gaspésie-îles-de-la-madeleine mental health budget | rss_11_mh_60 |
| rss 12 chaudière-appalaches mental health budget | rss_12_mh_3 |
| rss 12 chaudière-appalaches mental health budget | rss_12_mh_6 |
| rss 12 chaudière-appalaches mental health budget | rss_12_mh_12 |
| rss 12 chaudière-appalaches mental health budget | rss_12_mh_24 |
| rss 12 chaudière-appalaches mental health budget | rss_12_mh_36 |
| rss 12 chaudière-appalaches mental health budget | rss_12_mh_48 |
| rss 12 chaudière-appalaches mental health budget | rss_12_mh_60 |
| rss 13 laval mental health budget | rss_13_mh_3 |
| rss 13 laval mental health budget | rss_13_mh_6 |
| rss 13 laval mental health budget | rss_13_mh_12 |
| rss 13 laval mental health budget | rss_13_mh_24 |
| rss 13 laval mental health budget | rss_13_mh_36 |
| rss 13 laval mental health budget | rss_13_mh_48 |
| rss 13 laval mental health budget | rss_13_mh_60 |
| rss 14 lanaudière mental health budget | rss_14_mh_3 |
| rss 14 lanaudière mental health budget | rss_14_mh_6 |
| rss 14 lanaudière mental health budget | rss_14_mh_12 |
| rss 14 lanaudière mental health budget | rss_14_mh_24 |
| rss 14 lanaudière mental health budget | rss_14_mh_36 |
| rss 14 lanaudière mental health budget | rss_14_mh_48 |
| rss 14 lanaudière mental health budget | rss_14_mh_60 |
| rss 15 laurentides mental health budget | rss_15_mh_3 |
| rss 15 laurentides mental health budget | rss_15_mh_6 |
| rss 15 laurentides mental health budget | rss_15_mh_12 |
| rss 15 laurentides mental health budget | rss_15_mh_24 |
| rss 15 laurentides mental health budget | rss_15_mh_36 |
| rss 15 laurentides mental health budget | rss_15_mh_48 |
| rss 15 laurentides mental health budget | rss_15_mh_60 |
| rss 16 montérégie mental health budget | rss_16_mh_3 |
| rss 16 montérégie mental health budget | rss_16_mh_6 |
| rss 16 montérégie mental health budget | rss_16_mh_12 |
| rss 16 montérégie mental health budget | rss_16_mh_24 |
| rss 16 montérégie mental health budget | rss_16_mh_36 |
| rss 16 montérégie mental health budget | rss_16_mh_48 |
| rss 16 montérégie mental health budget | rss_16_mh_60 |
| rss 01 bas-saint-laurent addictions budget | rss_01_a_3 |
| rss 01 bas-saint-laurent addictions budget | rss_01_a_6 |
| rss 01 bas-saint-laurent addictions budget | rss_01_a_12 |
| rss 01 bas-saint-laurent addictions budget | rss_01_a_24 |
| rss 01 bas-saint-laurent addictions budget | rss_01_a_36 |
| rss 01 bas-saint-laurent addictions budget | rss_01_a_48 |
| rss 01 bas-saint-laurent addictions budget | rss_01_a_60 |
| rss 02 saguenay-lac-saint-jean addictions budget | rss_02_a_3 |
| rss 02 saguenay-lac-saint-jean addictions budget | rss_02_a_6 |
| rss 02 saguenay-lac-saint-jean addictions budget | rss_02_a_12 |
| rss 02 saguenay-lac-saint-jean addictions budget | rss_02_a_24 |
| rss 02 saguenay-lac-saint-jean addictions budget | rss_02_a_36 |
| rss 02 saguenay-lac-saint-jean addictions budget | rss_02_a_48 |
| rss 02 saguenay-lac-saint-jean addictions budget | rss_02_a_60 |
| rss 03 capitale-nationale addictions budget | rss_03_a_3 |
| rss 03 capitale-nationale addictions budget | rss_03_a_6 |
| rss 03 capitale-nationale addictions budget | rss_03_a_12 |
| rss 03 capitale-nationale addictions budget | rss_03_a_24 |
| rss 03 capitale-nationale addictions budget | rss_03_a_36 |
| rss 03 capitale-nationale addictions budget | rss_03_a_48 |
| rss 03 capitale-nationale addictions budget | rss_03_a_60 |
| rss 04 mauricie et centre-du-québec addictions budget | rss_04_a_3 |
| rss 04 mauricie et centre-du-québec addictions budget | rss_04_a_6 |
| rss 04 mauricie et centre-du-québec addictions budget | rss_04_a_12 |
| rss 04 mauricie et centre-du-québec addictions budget | rss_04_a_24 |
| rss 04 mauricie et centre-du-québec addictions budget | rss_04_a_36 |
| rss 04 mauricie et centre-du-québec addictions budget | rss_04_a_48 |
| rss 04 mauricie et centre-du-québec addictions budget | rss_04_a_60 |
| rss 05 estrie addictions budget | rss_05_a_3 |
| rss 05 estrie addictions budget | rss_05_a_6 |
| rss 05 estrie addictions budget | rss_05_a_12 |
| rss 05 estrie addictions budget | rss_05_a_24 |
| rss 05 estrie addictions budget | rss_05_a_36 |
| rss 05 estrie addictions budget | rss_05_a_48 |
| rss 05 estrie addictions budget | rss_05_a_60 |
| rss 06 montréal addictions budget | rss_06_a_3 |
| rss 06 montréal addictions budget | rss_06_a_6 |
| rss 06 montréal addictions budget | rss_06_a_12 |
| rss 06 montréal addictions budget | rss_06_a_24 |
| rss 06 montréal addictions budget | rss_06_a_36 |
| rss 06 montréal addictions budget | rss_06_a_48 |
| rss 06 montréal addictions budget | rss_06_a_60 |
| rss 07 outaouais addictions budget | rss_07_a_3 |
| rss 07 outaouais addictions budget | rss_07_a_6 |
| rss 07 outaouais addictions budget | rss_07_a_12 |
| rss 07 outaouais addictions budget | rss_07_a_24 |
| rss 07 outaouais addictions budget | rss_07_a_36 |
| rss 07 outaouais addictions budget | rss_07_a_48 |
| rss 07 outaouais addictions budget | rss_07_a_60 |
| rss 08 abitibi-témiscamingue addictions budget | rss_08_a_3 |
| rss 08 abitibi-témiscamingue addictions budget | rss_08_a_6 |
| rss 08 abitibi-témiscamingue addictions budget | rss_08_a_12 |
| rss 08 abitibi-témiscamingue addictions budget | rss_08_a_24 |
| rss 08 abitibi-témiscamingue addictions budget | rss_08_a_36 |
| rss 08 abitibi-témiscamingue addictions budget | rss_08_a_48 |
| rss 08 abitibi-témiscamingue addictions budget | rss_08_a_60 |
| rss 09 côte-nord addictions budget | rss_09_a_3 |
| rss 09 côte-nord addictions budget | rss_09_a_6 |
| rss 09 côte-nord addictions budget | rss_09_a_12 |
| rss 09 côte-nord addictions budget | rss_09_a_24 |
| rss 09 côte-nord addictions budget | rss_09_a_36 |
| rss 09 côte-nord addictions budget | rss_09_a_48 |
| rss 09 côte-nord addictions budget | rss_09_a_60 |
| rss 10 nord-du-québec addictions budget | rss_10_a_3 |
| rss 10 nord-du-québec addictions budget | rss_10_a_6 |
| rss 10 nord-du-québec addictions budget | rss_10_a_12 |
| rss 10 nord-du-québec addictions budget | rss_10_a_24 |
| rss 10 nord-du-québec addictions budget | rss_10_a_36 |
| rss 10 nord-du-québec addictions budget | rss_10_a_48 |
| rss 10 nord-du-québec addictions budget | rss_10_a_60 |
| rss 11 gaspésie--îles-de-la-madeleine addictions budget | rss_11_a_3 |
| rss 11 gaspésie--îles-de-la-madeleine addictions budget | rss_11_a_6 |
| rss 11 gaspésie--îles-de-la-madeleine addictions budget | rss_11_a_12 |
| rss 11 gaspésie--îles-de-la-madeleine addictions budget | rss_11_a_24 |
| rss 11 gaspésie--îles-de-la-madeleine addictions budget | rss_11_a_36 |
| rss 11 gaspésie--îles-de-la-madeleine addictions budget | rss_11_a_48 |
| rss 11 gaspésie--îles-de-la-madeleine addictions budget | rss_11_a_60 |
| rss 12 chaudière-appalaches addictions budget | rss_12_a_3 |
| rss 12 chaudière-appalaches addictions budget | rss_12_a_6 |
| rss 12 chaudière-appalaches addictions budget | rss_12_a_12 |
| rss 12 chaudière-appalaches addictions budget | rss_12_a_24 |
| rss 12 chaudière-appalaches addictions budget | rss_12_a_36 |
| rss 12 chaudière-appalaches addictions budget | rss_12_a_48 |
| rss 12 chaudière-appalaches addictions budget | rss_12_a_60 |
| rss 13 laval addictions budget | rss_13_a_3 |
| rss 13 laval addictions budget | rss_13_a_6 |
| rss 13 laval addictions budget | rss_13_a_12 |
| rss 13 laval addictions budget | rss_13_a_24 |
| rss 13 laval addictions budget | rss_13_a_36 |
| rss 13 laval addictions budget | rss_13_a_48 |
| rss 13 laval addictions budget | rss_13_a_60 |
| rss 14 lanaudière addictions budget | rss_14_a_3 |
| rss 14 lanaudière addictions budget | rss_14_a_6 |
| rss 14 lanaudière addictions budget | rss_14_a_12 |
| rss 14 lanaudière addictions budget | rss_14_a_24 |
| rss 14 lanaudière addictions budget | rss_14_a_36 |
| rss 14 lanaudière addictions budget | rss_14_a_48 |
| rss 14 lanaudière addictions budget | rss_14_a_60 |
| rss 15 laurentides addictions budget | rss_15_a_3 |
| rss 15 laurentides addictions budget | rss_15_a_6 |
| rss 15 laurentides addictions budget | rss_15_a_12 |
| rss 15 laurentides addictions budget | rss_15_a_24 |
| rss 15 laurentides addictions budget | rss_15_a_36 |
| rss 15 laurentides addictions budget | rss_15_a_48 |
| rss 15 laurentides addictions budget | rss_15_a_60 |
| rss 16 montérégie addictions budget | rss_16_a_3 |
| rss 16 montérégie addictions budget | rss_16_a_6 |
| rss 16 montérégie addictions budget | rss_16_a_12 |
| rss 16 montérégie addictions budget | rss_16_a_24 |
| rss 16 montérégie addictions budget | rss_16_a_36 |
| rss 16 montérégie addictions budget | rss_16_a_48 |
| rss 16 montérégie addictions budget | rss_16_a_60 |
| regional mental health budget ($/capita) | region_mhbudget_2018-2019 |
| regional mental health budget ($/capita) | region_mhbudget_2017-2018 |
| regional mental health budget ($/capita) | region_mhbudget_2016-2017 |
| regional mental health budget ($/capita) | region_mhbudget_2015-2016 |
| regional addictions budget ($/capita) | region_abudget_2018-2019 |
| regional addictions budget ($/capita) | region_abudget_2017-2018 |
| regional addictions budget ($/capita) | region_abudget_2016-2017 |
| regional addictions budget ($/capita) | region_abudget_2015-2016 |

# Quality of Care Indicators (quality care)

- quality of anxiety or depressive disorders mental health services follow-up in primary care (continuous)
- quality of mental health services depression disorder mental health services follow-up in primary care (continuous)
- quality of substance use disorder mental health services follow-up in primary care (continuous)
- quality of mental health care services follow-up after hospitalization: readmission within 30 days (continuous)
- quality of mental health services follow-up in primary care after suicide attempt (continuous)
- quality of community mental health services (continuous)
- quality of community mental health services of patients with severe mental illness (continuous)
- quality of community mental health services of patients with common mental disorders (continuous)
- quality of community mental health services of patients with personality disorders (continuous)
- adequate use of emergency room for mental health service (continuous)

| derived variables | variable name |
| --- | --- |
| quality of anxiety or depressive disorders mental health services follow-up in primary care (continuous) | qfu_primcare_anxdep_3 |
| quality of anxiety or depressive disorders mental health services follow-up in primary care (continuous) | qfu_primcare_anxdep_6 |
| quality of anxiety or depressive disorders mental health services follow-up in primary care (continuous) | qfu_primcare_anxdep_12 |
| quality of anxiety or depressive disorders mental health services follow-up in primary care (continuous) | qfu_primcare_anxdep_24 |
| quality of anxiety or depressive disorders mental health services follow-up in primary care (continuous) | qfu_primcare_anxdep_36 |
| quality of anxiety or depressive disorders mental health services follow-up in primary care (continuous) | qfu_primcare_anxdep_48 |
| quality of anxiety or depressive disorders mental health services follow-up in primary care (continuous) | qfu_primcare_anxdep_60 |
| quality of depression disorder mental health services follow-up in primary care (continuous) | qfu_primcare_dep_3 |
| quality of depression disorder mental health services follow-up in primary care (continuous) | qfu_primcare_dep_6 |
| quality of depression disorder mental health services follow-up in primary care (continuous) | qfu_primcare_dep_12 |
| quality of depression disorder mental health services follow-up in primary care (continuous) | qfu_primcare_dep_24 |
| quality of depression disorder mental health services follow-up in primary care (continuous) | qfu_primcare_dep_36 |
| quality of depression disorder mental health services follow-up in primary care (continuous) | qfu_primcare_dep_48 |
| quality of depression disorder mental health services follow-up in primary care (continuous) | qfu_primcare_dep_60 |
| quality of substance use disorder mental health services follow-up in primary care (continuous) | qfu_primcare_sud_3 |
| quality of substance use disorder mental health services follow-up in primary care (continuous) | qfu_primcare_sud_6 |
| quality of substance use disorder mental health services follow-up in primary care (continuous) | qfu_primcare_sud_12 |
| quality of substance use disorder mental health services follow-up in primary care (continuous) | qfu_primcare_sud_24 |
| quality of substance use disorder mental health services follow-up in primary care (continuous) | qfu_primcare_sud_36 |
| quality of substance use disorder mental health services follow-up in primary care (continuous) | qfu_primcare_sud_48 |
| quality of substance use disorder mental health services follow-up in primary care (continuous) | qfu_primcare_sud_60 |
| quality of mental health care services follow-up after hospitalization: readmission within 30 days (continuous) | qfu_posthosp_readmit30_3 |
| quality of mental health care services follow-up after hospitalization: readmission within 30 days (continuous) | qfu_posthosp_readmit30_6 |
| quality of mental health care services follow-up after hospitalization: readmission within 30 days (continuous) | qfu_posthosp_readmit30_12 |
| quality of mental health care services follow-up after hospitalization: readmission within 30 days (continuous) | qfu_posthosp_readmit30_24 |
| quality of mental health care services follow-up after hospitalization: readmission within 30 days (continuous) | qfu_posthosp_readmit30_36 |
| quality of mental health care services follow-up after hospitalization: readmission within 30 days (continuous) | qfu_posthosp_readmit30_48 |
| quality of mental health care services follow-up after hospitalization: readmission within 30 days (continuous) | qfu_posthosp_readmit30_60 |
| quality of mental health services followup in primary care after suicide attempt (continuous) | qfu_primcare_postsuicideattempt_3 |
| quality of mental health services followup in primary care after suicide attempt (continuous) | qfu_primcare_postsuicideattempt_6 |
| quality of mental health services followup in primary care after suicide attempt (continuous) | qfu_primcare_postsuicideattempt_12 |
| quality of mental health services followup in primary care after suicide attempt (continuous) | qfu_primcare_postsuicideattempt_24 |
| quality of mental health services followup in primary care after suicide attempt (continuous) | qfu_primcare_postsuicideattempt_36 |
| quality of mental health services followup in primary care after suicide attempt (continuous) | qfu_primcare_postsuicideattempt_48 |
| quality of mental health services followup in primary care after suicide attempt (continuous) | qfu_primcare_postsuicideattempt_60 |
| quality of community mental health services (continuous) | qcomserv_3 |
| quality of community mental health services (continuous) | qcomserv_6 |
| quality of community mental health services (continuous) | qcomserv_12 |
| quality of community mental health services (continuous) | qcomserv_24 |
| quality of community mental health services (continuous) | qcomserv_36 |
| quality of community mental health services (continuous) | qcomserv_48 |
| quality of community mental health services (continuous) | qcomserv_60 |
| quality of community mental health services of patients with severe mental illness (continuous) | qcomserv_severe_3 |
| quality of community mental health services of patients with severe mental illness (continuous) | qcomserv_severe_6 |
| quality of community mental health services of patients with severe mental illness (continuous) | qcomserv_severe_12 |
| quality of community mental health services of patients with severe mental illness (continuous) | qcomserv_severe_24 |
| quality of community mental health services of patients with severe mental illness (continuous) | qcomserv_severe_36 |
| quality of community mental health services of patients with severe mental illness (continuous) | qcomserv_severe_48 |
| quality of community mental health services of patients with severe mental illness (continuous) | qcomserv_severe_60 |
| quality of community mental health services of patients with common mental disorders (continuous) | qcomserv_cmd_3 |
| quality of community mental health services of patients with common mental disorders (continuous) | qcomserv_cmd_6 |
| quality of community mental health services of patients with common mental disorders (continuous) | qcomserv_cmd_12 |
| quality of community mental health services of patients with common mental disorders (continuous) | qcomserv_cmd_24 |
| quality of community mental health services of patients with common mental disorders (continuous) | qcomserv_cmd_36 |
| quality of community mental health services of patients with common mental disorders (continuous) | qcomserv_cmd_48 |
| quality of community mental health services of patients with common mental disorders (continuous) | qcomserv_cmd_60 |
| quality of community mental health services of patients with personality disorders (continuous) | qcomserv_pd_3 |
| quality of community mental health services of patients with personality disorders (continuous) | qcomserv_pd_6 |
| quality of community mental health services of patients with personality disorders (continuous) | qcomserv_pd_12 |
| quality of community mental health services of patients with personality disorders (continuous) | qcomserv_pd_24 |
| quality of community mental health services of patients with personality disorders (continuous) | qcomserv_pd_36 |
| quality of community mental health services of patients with personality disorders (continuous) | qcomserv_pd_48 |
| quality of community mental health services of patients with personality disorders (continuous) | qcomserv_pd_60 |
| adequate use of emergency room for mental health service (continuous) | aduse_er_3 |
| adequate use of emergency room for mental health service (continuous) | aduse_er_6 |
| adequate use of emergency room for mental health service (continuous) | aduse_er_12 |
| adequate use of emergency room for mental health service (continuous) | aduse_er_24 |
| adequate use of emergency room for mental health service (continuous) | aduse_er_36 |
| adequate use of emergency room for mental health service (continuous) | aduse_er_48 |
| adequate use of emergency room for mental health service (continuous) | aduse_er_60 |
