# Supplementary material for: Explainable artificial intelligence models for predicting risk of suicide using health administrative data in Quebec
Source: PLoS One. 2024 Apr 3;19(4):e0301117. doi: 10.1371/journal.pone.0301117 (PMC10990247; doi:10.1371/journal.pone.0301117)
Supplement: S2 Table — (DOCX) [file pone.0301117.s002.docx]

**S 2 Table: Hyperparameters**

| Classification model | Tunde hyperparameters |
| --- | --- |
| LR | Penalty: L1 ,L2 , solver: saga, lbfgs |
| RF | n_estimators :200,300 , max tree depth =5 |
| XGBoost | max tree depth =3,5 ,gamma :0.1,0.2 minimum child weight=5, subsample ratio of columns = 0.5,0.8,0.9 |
| MLP | Activation: relu,logistic  Alpha :0.0001,0.001  Hidden layer sizes: [(8,), (15,)]  Solver: Adam  Learning rate: adaptive  Max iter: [50, 150] |

LR: Logistic Regression; RF: Random Forest; XGBoost: Extreme Gradient Boosting; MLP Multilayer perceptron
