# Supplementary material for: Explainable artificial intelligence models for predicting risk of suicide using health administrative data in Quebec
Source: PLoS One. 2024 Apr 3;19(4):e0301117. doi: 10.1371/journal.pone.0301117 (PMC10990247; doi:10.1371/journal.pone.0301117)
Supplement: S1 Fig — (DOCX) [file pone.0301117.s003.docx]

**S 3 Fig: Reliability diagram before and after calibration**

**
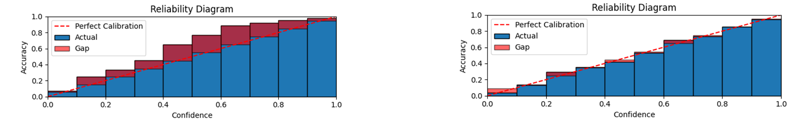
**

**
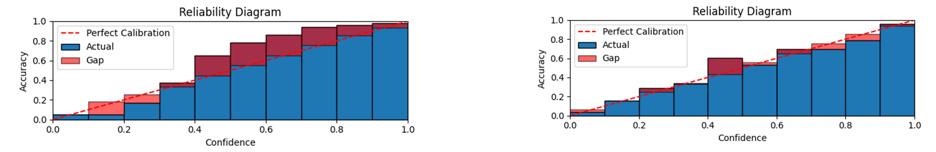
**

**
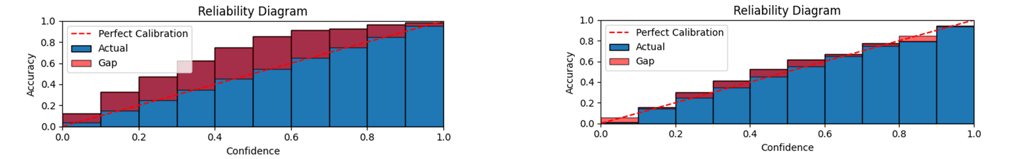
**

LR: Logistic Regression; RF: Random Forest; XGBoost: Extreme Gradient Boosting; MLP Multilayer perceptron
